# Synthetic Gene Circuits for Regulation of Next‐Generation Cell‐Based Therapeutics

**DOI:** 10.1002/advs.202309088

**Published:** 2023-12-21

**Authors:** Ana P. Teixeira, Martin Fussenegger

**Affiliations:** ^1^ Department of Biosystems Science and Engineering ETH Zurich Klingelbergstrasse 48 Basel CH‐4056 Switzerland; ^2^ Faculty of Science University of Basel Klingelbergstrasse 48 Basel CH‐4056 Switzerland

**Keywords:** cell‐based therapies, gene control, gene switches, gene therapy, synthetic biology

## Abstract

Arming human cells with synthetic gene circuits enables to expand their capacity to execute superior sensing and response actions, offering tremendous potential for innovative cellular therapeutics. This can be achieved by assembling components from an ever‐expanding molecular toolkit, incorporating switches based on transcriptional, translational, or post‐translational control mechanisms. This review provides examples from the three classes of switches, and discusses their advantages and limitations to regulate the activity of therapeutic cells in vivo. Genetic switches designed to recognize internal disease‐associated signals often encode intricate actuation programs that orchestrate a reduction in the sensed signal, establishing a closed‐loop architecture. Conversely, switches engineered to detect external molecular or physical cues operate in an open‐loop fashion, switching on or off upon signal exposure. The integration of such synthetic gene circuits into the next generation of chimeric antigen receptor T‐cells is already enabling precise calibration of immune responses in terms of magnitude and timing, thereby improving the potency and safety of therapeutic cells. Furthermore, pre‐clinical engineered cells targeting other chronic diseases are gathering increasing attention, and this review discusses the path forward for achieving clinical success. With synthetic biology at the forefront, cellular therapeutics holds great promise for groundbreaking treatments.

## Graphical abstract

This review details examples of synthetic gene circuits based on transcriptional, translational, and post‐translational control mechanisms and discusses how they can be used for conditional regulation of protein expression and/or activity in response to specific internal or external stimuli, thereby enhancing the therapeutic efficacy and safety of engineered cell therapies.

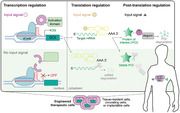

## 1 Introduction

Living cells encode an intricate armament of sensors, actuators, and regulators dedicated to maintaining intracellular homeostasis. Building upon these inherent sensory and biosynthetic capabilities, human cells have been engineered with synthetic devices that enable them to actively sense specific extracellular stimuli and orchestrate desired therapeutic responses. These living therapeutics are rapidly becoming the cornerstone of modern personalized medicine.^[^ ^1^ ^]^ The incorporation of synthetic gene circuits into human cells is allowing precise control over the timing and dosage of therapeutic actions, thereby enhancing the safety and efficacy of gene‐ and cell‐based therapies. These circuits can operate either as open‐loop systems, designed to respond to user‐defined exogenous molecular or physical signals, or closed‐loop systems, capable of autonomously detecting molecular inputs and initiating downstream processes.^[^ ^2^ ^]^ Closed‐loop circuits often incorporate natural or chimeric cell surface receptors which detect specific disease‐associated small molecules or proteins, whether soluble or present on the membrane of target cells. In response, signaling cascades are initiated, culminating in tailored therapeutic actions aimed at maintaining the signal within a normal physiological range.^[^ ^3^, ^4^, ^5^ ^]^

External molecular signals primarily involve small molecules,^[^ ^6^, ^7^, ^8^, ^9^, ^10^ ^]^ but also extend to oligonucleotides^[^ ^11^ ^]^ and peptides or proteins,^[^ ^12^, ^13^ ^]^ while physical signals span light of various wavelengths,^[^ ^14^, ^15^, ^16^ ^]^ electrical currents,^[^ ^17^, ^18^ ^]^ temperature variations,^[^ ^19^, ^20^ ^]^ and mechanical forces.^[^ ^21^, ^22^ ^]^ Within the realm of molecular signals, small molecules offer several advantages, including oral administration, lower production costs, good cell membrane permeability, and immune system evasion. A wide range of small molecules have been used to control protein or mRNA levels in human cells, including ligands for natural or chimeric receptors,^[^ ^5^ ^]^ ligands for bacterial transcription factors (TFs),^[^ ^6^, ^7^, ^9^ ^]^ protein dimerization inducers,^[^ ^23^, ^24^ ^]^ protein degradation inducers,^[^ ^25^, ^26^ ^]^ riboswitch modulators,^[^ ^27^, ^28^, ^29^ ^]^ and viral protease inhibitors.^[^ ^8^, ^30^, ^31^ ^]^ Conversely, actuation via physical signals can also offer several benefits for in vivo regulation of protein expression/activity. These include non‐invasiveness for enhanced patient comfort, precise spatial control for targeting specific areas or tissues, and the capacity to prompt rapid cellular responses, rendering physical signals particularly suitable for real‐time adjustments and immediate therapeutic actions. Furthermore, unlike some molecular signals, physical signals are less susceptible to cellular metabolism and degradation.

**Figure 1:**
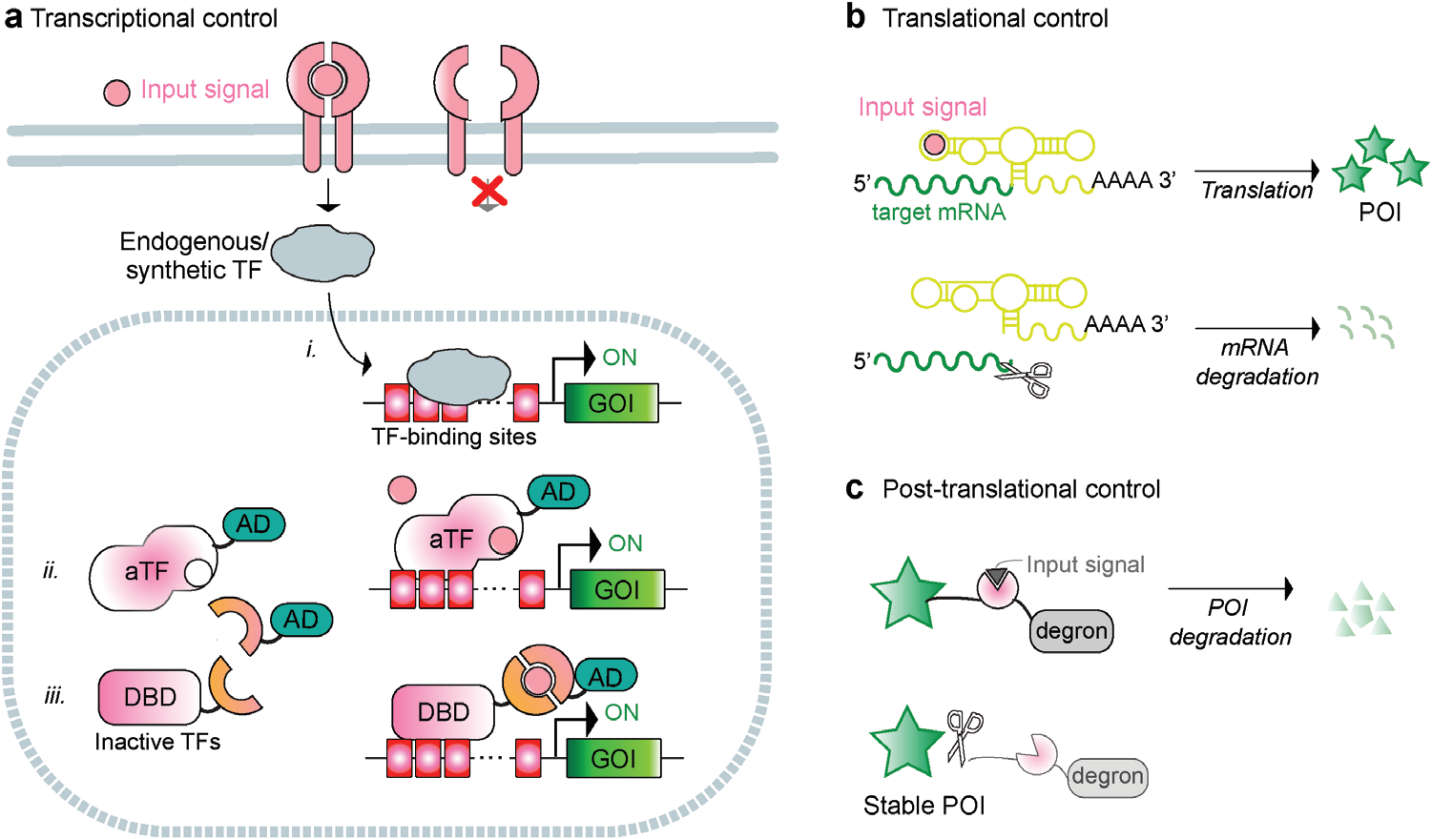
Multi‐level regulation of protein expression via small molecules or physical signals. a) Transcriptional control. i. The signal input activates cell surface receptors or channels, which ultimately activate endogenous or synthetic transcription factors that regulate the transcription of the target gene (either from a native locus, or from an artificial locus introduced in the host cell genome). ii‐iii. The signal input can also directly control the activity of a transcription regulator. ii. The transcription regulator can consist of a natural DBD and a ligand‐binding domain, in which the ligand (signal input) controls the interaction of the allosteric transcription factor (aTF) with specific DNA sequences. In the example shown, aTF becomes DNA‐binding–competent in the presence of the signal input. When fused to a mammalian activation domain (AD), this triggers the expression of the gene of interest (GOI). iii. Alternatively, split transcription regulators, consisting of a synthetic DBD and a regulatory domain, each fused to complementary pairs of inducible dimerizing partners, can be assembled in the presence of the dimerizing signal to control the expression of the GOI. b) Translation control. The target mRNA contains elements in the 5′ or 3′ untranslated regions (UTR) which regulate the expression of the protein of interest (POI) by binding a small molecule, causing a change in their structural conformation. In the example shown, the signal input inhibits an aptazyme in the 3′ UTR which cannot self‐cleave, therefore stabilizing the target mRNA for translation into the POI. c) Post‐translational control. Regulation of protein activity using external signals can be achieved by fusing destabilizing domains (or degrons) and inhibitable proteases. In the example shown, the POI is fused to a protease and a degron via a protease cleavage site. At baseline, the degron module undergoes self‐cleavage, which stabilizes the POI. However, in the presence of a protease inhibitor, the degron remains attached to the POI, leading to its degradation.

Many synthetic gene circuits rely primarily on transcriptional control, which involves either direct activation of transcription effectors by the trigger signal, or indirect activation through signal detection by transmembrane sensors, resulting in signal propagation to the nucleus (**Figure** **1**a).^[^ ^3^ ^]^ Nevertheless, synthetic devices based on translational (Figure 1b) or post‐translational (Figure 1c) control mechanisms have attracted increasing interest in recent years.^[^ ^32^, ^34^ ^]^ While transcriptional control typically affords wide dynamic ranges and high activation levels, post‐transcriptional circuits offer distinct benefits, including faster response times and the ability to be delivered as RNA, thereby avoiding safety concerns related to genomic integration. Here we present examples of engineered genetic circuits from all control classes and discuss how they have been used for conditional regulation of protein expression and/or activity in response to specific stimuli, thereby enhancing the therapeutic efficacy and safety of cell‐based therapies. Particular emphasis is placed on how they are facilitating the clinical translation of chimeric antigen receptor (CAR)T cells targeting a range of tumors, as well as potential breakthroughs that lie ahead in the treatment of chronic diseases.

## 2 Natural Transmembrane Protein Sensors Orchestrating Transcription Regulation

**Figure 2:**
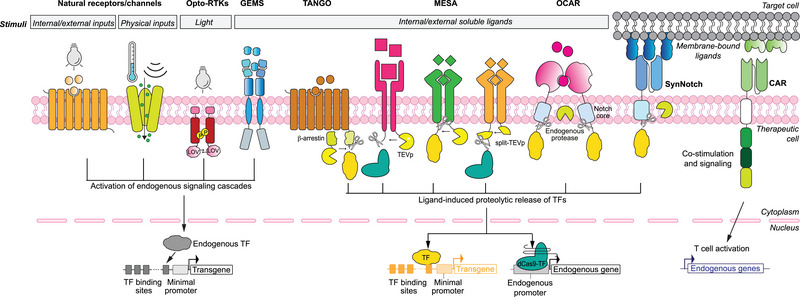
Transcriptional control following signal detection by native or chimeric cell surface receptors or channels. To regulate the expression of transgenes or endogenous genes by extracellular signals, endogenous or synthetic transcription regulators activated by constitutively expressed natural or synthetic sensor proteins located at the plasma membrane need to be targeted to the promoter region of the GOI. To target endogenous genes, dCas9‐based transcription factors are usually employed. Illustrated are receptor platforms that sense internal (soluble or membrane‐bound) or external (molecular or physical) signal inputs, and rely on either endogenous signaling cascades (Opto‐RTKs, GEMS, and CARs) or synthetic TFs which are proteolytically released upon receptor signal detection (TANGO, MESA, OCAR, and synNotch). See the text for a detailed description of the molecular parts.

Plasma membrane receptors and channels have been employed in mammalian synthetic biology to regulate the transcription of transgenes in response to diverse classes of ligands and other external signals. Notable examples include the large family of G‐protein–coupled receptors (GPCRs), which play crucial roles in mediating cellular responses to diverse extracellular stimuli, including proteins, small peptides, metabolites, light, ions, etc. Their ability to detect and respond to specific cues by triggering second messenger molecules that activate and orchestrate intracellular signaling pathways makes them attractive candidates for engineering mammalian genetic switches. Target transgenes are placed downstream of DNA response elements specific to the transcriptional effector of the endogenous signaling cascade triggered upon receptor activation (**Figure** **2**). This approach has been applied to engineer mammalian cells for sensing histamine levels characteristic of allergies (via the histamine HRH2 receptor) or elevated bile acid levels (via the TGR5 receptor) indicative of impaired liver function.^[^ ^5^, ^35^ ^]^ These receptors utilize the cAMP signaling pathway, which can be rewired to trigger desired transcriptional responses, thereby effectively regulating the elevated levels of the targeted sensing molecules in a closed‐loop manner. In addition to small‐molecule–responsive GPCRs, light‐sensitive GPCRs such as the human melanopsin receptor (OPN4) naturally expressed by retinal ganglion cells have been harnessed to achieve precise spatiotemporal control over cellular behavior in response to light. By rewiring the blue‐light–induced intracellular calcium increase in OPN4‐expressing HEK cells to transcription activation from synthetic promoters responsive to nuclear factor of activated T‐cells (NFAT), downstream transgenes can be regulated in response to light irradiation.^[^ ^15^ ^]^

Moreover, both human and bacterial channels have been explored as mediators to equip cells with new sensory capabilities. For instance, the integration of a voltage‐gated calcium channel into HEK cells enabled the initiation of native calcium signaling upon encountering elevated glucose levels. By using NFAT‐responsive promoters, transgene expression was effectively triggered under hyperglycemic conditions.^[^ ^36^ ^]^ Similarly, the human non‐selective cation channel transient receptor potential melastatin 8, which can mediate sustained calcium influx upon activation, has been employed to engineer cells that regulate therapeutic gene expression in response to menthol or exposure to cool temperatures.^[^ ^10^ ^]^ When such engineered cells were microencapsulated and implanted under the skin of mice, transdermal delivery of menthol was able to alleviate hyperglycemia in a model of type 1 diabetes or to reverse experimental muscle atrophy. Moreover, T cells ectopically expressing the human mechanosensitive Piezo1 ion channel can be effectively stimulated by ultrasound waves when surrounded by extracellular microbubbles, leading to increased transcriptional activation from NFAT promoters.^[^ ^37^ ^]^ In a recent study, human cells genetically modified with the bacterial large conductance mechanosensitive channel MscL were sensitized to respond to sound waves. Music‐actuated mechanical forces imposed in the cell membrane cause MscL channel opening, thereby leading to intracellular calcium increase and release of protein‐containing vesicles.^[^ ^21^ ^]^

## 3 Designer Transmembrane Receptors Orchestrating Transcription Regulation

Natural cell membrane receptors have been modified to couple ligand binding with synthetic orthogonal downstream signaling events. This enables the design of synthetic circuits that do not interfere with endogenous cellular processes. Among the earliest approaches for the development of chimeric receptors is the Tango system, which combines natural or evolved sensing modules with synthetic protease‐based signal transduction modules.^[^ ^38^, ^39^ ^]^ Intracellularly, Tango receptors have an attached synthetic TF which is released via tobacco etch virus protease (TEVp) cleavage upon ligand binding to the receptor. TEVp is fused to a signaling protein (β‐arrestin) which is recruited upon receptor activation. The released TF can then enter the nucleus and activate the expression of user‐specified genes (Figure 2). This approach has been applied to reprogram various receptors, including GPCRs, receptor tyrosine kinases (RTKs), and steroid hormone receptors, in order to induce transgene expression in response to synthetic small molecules, hormones, mitogens, chemokines, and fatty acids.^[^ ^39^, ^40^ ^]^

The rewiring of aberrant signaling to effector release (RASER) system also relies on proteolytic release of effector proteins upon sustained activation of native RTK receptors.^[^ ^41^ ^]^ Oncogenic overactivation of RTK signaling through ERBB receptors (such as epidermal growth factor receptor) activates RASER synthetic signaling cascades, which consist of two chimeric signaling proteins that are recruited to bind to phosphotyrosine sites on activated ERBB receptors. One signaling protein is linked to a protease, while the other is connected to a cleavable effector protein, which was shown to be released in cancer cell lines with hyperactive ERBB signaling. This system could induce cell death through the activation of pro‐apoptotic proteins when delivered to cancer cell lines with sustained signaling over time, leaving normal cells unaffected.

Nevertheless, certain molecules acting as disease biomarkers are not recognized by any known natural receptor. To broaden the spectrum of detectable molecules, cells have been genetically engineered to express synthetic receptors featuring extracellular domains composed of single‐chain fragment variable (scFv) antibodies or other ligand‐binding domains targeting small‐molecules or protein‐based antigens.^[^ ^42^, ^43^, ^44^ ^]^ The modular extracellular sensor architecture (MESA) is one of such platforms. MESA receptors also retain TFs at the cell membrane and release them when activated by soluble ligands through TEVp cleavage. However, MESA receptors depend on ligand‐induced receptor chain dimerization, with one chain linked to the transcription factor via a TEVp cleavage site, while the other chain recruits the TEV protease. An improved configuration employs split TEVp fragments rationally mutated to minimize their spontaneous reconstitution, affording a low background in the absence of the receptor ligand and high fold‐induction of reporter expression when the ligand is present.^[^ ^45^ ^]^ MESA and Tango receptors have been adapted to regulate endogenous gene expression by incorporating nulease‐deficient Cas9 (dCas9) fused to a transcriptional activation domain (AD).^[^ ^40^, ^46^ ^]^ When a ligand binds to the receptor, it triggers the release of the dCas9–AD complex, which then associates with the sgRNA, allowing specific and multiple loci to be targeted for gene expression activation. These approaches utilize rewiring of receptors from both GPCR and RTK classes to gradually increase expression levels of target genes in an agonist dose‐dependent manner, using a variety of soluble ligands (including proteins, lipids, and sugars).

Additionally, RTKs have been engineered to include light‐oxygen–voltage (LOV) domains from various organisms in their cytosolic tail. These modified receptors, known as opto‐RTKs,^[^ ^47^ ^]^ homodimerize and become activated in response to blue‐light exposure, triggering the respective endogenous downstream signaling pathways (Figure 2) with high spatiotemporal precision. Red‐light‐responsive RTKs have been also engineered, for instance taking advantage of a domain derived from the *Synechocystis* cyanobacterial phytochrome 1, which homodimerizes upon illumination with red light.^[^ ^48^ ^]^ Transcriptional activation reporters of the signaling pathways afforded increased expression under red‐light exposure versus dark conditions.

The generalized extracellular molecule sensor (GEMS) platform leverages the signal amplification achieved with native signaling cascades, while endowing cells with novel sensing capabilities. It employs the erythropoietin receptor fused to different ligand‐binding domains for sensing a wide range of extracellular soluble small molecules and proteins (including disease‐relevant markers, such as prostate‐specific antigen and thrombotic fibrin degradation products).^[^ ^42^, ^49^, ^50^ ^]^ The cytoplasmic domain of GEMS receptors can be swapped for activation of four endogenous signaling pathways (JAK/STAT, ERK/MAPK, phospholipase C, and PI3K/AKT) to concomitantly drive user‐defined transcriptional programs. High fold‐induction rates of gene expression are achieved upon ligand‐mediated receptor dimerization. The scope of GEMS was later expanded to enable the rewiring of endogenous pathways upon synthetic receptor activation for endogenous gene regulation.^[^ ^51^ ^]^ This was achieved by fusing the MS2 bacteriophage coat protein (MCP) to transcriptional regulators from the endogenous NFAT, NFκB, MAPK, or SMAD pathways, and rerouting the activation of these pathways to dCas9‐directed gene expression from genomic loci via a guide RNA containing MS2 loops. This system enabled dose‐dependent IL‐12 production in response to the immunomodulatory cytokines TGFβ and TNFα.^[^ ^51^ ^]^

Another group of chimeric receptors are the SynNotch receptors.^[^ ^43^ ^]^ They contain a customizable extracellular antigen‐binding domain and an intracellular synthetic TF, most commonly the yeast‐ and herpes‐virus–derived Gal4‐VP64. The two are linked by a transmembrane domain from the Notch‐1 protein. Engagement of membrane‐bound antigens with the extracellular domain leads to cleavage of the transmembrane domain by a γ‐secretase enzyme, releasing the transcription factor, which translocates to the nucleus, binds to Gal4‐recognition DNA sites, and promotes expression of the downstream transgene. The synNotch platform has been applied in many studies, mainly to respond to sender cells bearing the target antigens in their plasma membrane,^[^ ^4^, ^52^, ^53^, ^54^ ^]^ but also to detect antigens exposed on enveloped viral particles.^[^ ^55^ ^]^ Furthermore, it has also recently been modified into orthogonal chemically activated cell‐surface receptors (OCARs), which respond to small molecule inducers of dimerization^[^ ^56^ ^]^ (Figure 2). Nevertheless, the original synNotch receptor has limitations that hinder its clinical translation, including the use of non‐human components that could elicit immune rejection, and high background activity in the off state. To overcome these limitations, the same research group recently introduced synthetic intramembrane proteolysis receptors (SNIPRs), featuring optimized extracellular, transmembrane, and juxtamembrane domains to maximize ligand‐dependent cleavage and human synthetic transcription factors to minimize immunogenicity.^[^ ^57^ ^]^ SNIPR with the DNA‐binding domain (DBD) from the liver‐specific human transcription factor hepatocyte nuclear factor 1‐alpha linked to the human p65 transactivation domain demonstrated superior performance in response to antigen binding. This human SNIPR was then used to activate the expression of a CAR and the resulting dual‐antigen–sensing circuit was tested in mouse‐tumor models, enabling specific eradication of cells expressing both antigens.

Chimeric antigen receptors expressed in immune T cells combine engineered sensing directed at cancer biomarkers with natural T cell actuation signals. CARs predominantly feature the extracellular domain in the form of scFvs of monoclonal antibodies that selectively bind to cancer‐specific antigens. Additionally, the potential of designed ankyrin repeat proteins (DARPins) to bind antigens with high affinity has also been explored in CARs.^[^ ^58^ ^]^ Upon target engagement, the intracellular CAR domains (e.g., CD3ζ, CD28, and 4‐1BB) activate signaling pathways that stimulate the T cell to produce co‐stimulatory signals, promoting T cell function, proliferation, and survival. This results in the killing of cancer cells expressing the targeted molecule. As we will discuss later, CAR‐T cells achieved a significant milestone as the first synthetic biology‐based therapies to obtain clinical approval for the treatment of blood cancers, and ongoing engineering efforts continue to enhance their therapeutic potential for treating solid tumors and other diseases.

Whether by leveraging the inherent properties of natural receptors or creating new functionalities through chimeric receptor designs, a variety of regulatory systems exists to finely modulate the transcriptional activity of selected therapeutic programs with high precision and specificity.

## 4 Transcription Regulation Relying on Natural DNA‐Binding Domains

A wide variety of bacterial TFs have been ported to mammalian cells to develop synthetic gene switches that regulate transgene expression in response to small molecules or other environmental signals. TFs responsive to antibiotics (e.g., tetracycline^[^ ^59^ ^]^), sugars (e.g., xylose^[^ ^7^ ^]^), or other plant‐derived compounds (e.g., vanillic acid,^[^ ^60^ ^]^ ferulic acid,^[^ ^61^ ^]^ phloretin,^[^ ^62^ ^]^ and protocatechuic acid^[^ ^63^ ^]^), amino acids (e.g., tryptophan^[^ ^64^ ^]^ and arginine^[^ ^65^ ^]^), fatty acids,^[^ ^66^ ^]^ intermediates of microbial carbohydrate metabolism (e.g., acetoin^[^ ^9^ ^]^ and gluconate^[^ ^6^ ^]^), vitamins (e.g., biotin^[^ ^67^ ^]^), temperature changes,^[^ ^19^, ^68^ ^]^ or light,^[^ ^69^ ^]^ are widespread throughout the bacterial kingdom, being involved in the regulation of many metabolic processes. These TFs generally possess a DBD, the most common consisting of a helix‐turn‐helix (HTH) motif, responsible for recognition and binding to specific DNA sequences known as operator sites. Additionally, they have a ligand‐binding domain (LBD), responsible for binding the effector molecule. The binding of the inducer triggers conformational changes in the TF which can have different effects, either enhancing its DNA‐binding affinity or preventing the TF from binding to DNA. Most microbial HTH transcriptional regulators bind DNA as homodimers.^[^ ^70^ ^]^

**Figure 3:**
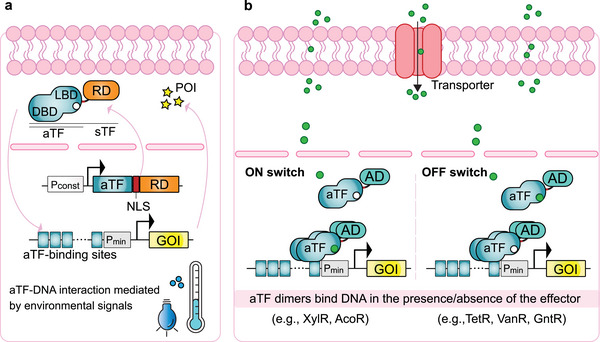
Microbial‐derived transcription factors for gene expression regulation in mammalian cells. a) Allosteric transcription factors (aTF) composed of a DNA‐binding domain (DBD) and a ligand‐binding domain (LBD) interact with their cognate DNA‐binding sequences in response to a diverse range of environmental signals, such as carbohydrates, amino acids, short‐chain carboxylic acids, vitamins, and light of different wavelength. To build mammalian gene switches responsive to these external stimuli, the aTFs fused to mammalian regulatory domains (RD) are constitutively expressed and the GOI is placed downstream of a synthetic promoter containing tandem repeats of the aTF binding sites. The resulting synthetic TF (sTF) can also include nuclear localization signals (NLS) to promote translocation to the nucleus. b) Examples of bacterial aTFs which have been harnessed to build mammalian gene switches. aTFs usually bind the target DNA as dimers. When exposed to their respective ligands, aTFs fused to activation domains (AD) can bind to the target DNA and activate transcription of the GOI (e.g., the xylose or acetoin‐responsive TFs, XylR, and AcoR). Alternatively, ligand‐bound aTFs cannot interact with their cognate DNA‐binding motifs, thereby turning off transcription (e.g., the tetracycline [TetR], vanillic acid [VanR], or gluconate [GntR] TFs). Most molecular signals can freely cross the mammalian plasma membrane, but dedicated transporters can be co‐expressed to enhance the sensitivity of the gene switches.

The use of bacterial TFs in mammalian cells requires careful consideration due to the differences in cellular machinery, often necessitating modifications with appropriate domains to ensure efficient transcriptional regulation and nuclear localization. To facilitate transport into the nucleus, nuclear localization signals (NLS) can be added to the protein sequence, while fusion with mammalian‐specific activation or repression domains (Box 1) can enhance their transcriptional activation or suppression (**Figure** **3**a). Furthermore, the operator sequences are positioned in the promoter region of the target genes, often in tandem repeats with an optimized spacer sequence between them, to attain substantial fold‐changes and wide dynamic ranges of expression.

**Box 1. Effectors of Gene Regulation**

As transactivation domains, viral VP16 derived from herpes simplex virus, its tetramer VP64, and Rta from Epstein–Barr virus are widely used in combination with various DNA‐binding domains to activate gene expression from targeted promoters.^[^ ^185^ ^]^ They effectively recruit components of the RNA polymerase II machinery upon binding to the promoter region of a target gene. This process leads to an increased rate of transcription initiation and subsequently boosts gene expression. The p65 subunit of the human NF‐κB complex is also commonly used as a transcription activator, being the preferred domain for developing humanized gene regulation tools.^[^ ^57^ ^]^ The tandem fusion of VP64, p65, and Rta, known as VPR, has also been extensively employed due to its enhanced activity.^[^ ^186^ ^]^ However, its larger size compared to VP16/VP64 poses challenges for incorporation into fusion proteins or delivery systems.

The most common trans‐silencer domains are Krüppel‐associated box (KRAB) domains from zinc finger proteins.^[^ ^187^ ^]^ These domains repress transcription via recruitment of corepressors, including histone deacetylases. When designed to bind the DNA near the transcriptional start site of a gene, the DNA‐binding modules (ZFs, transcriptional activator‐like effectors [TALEs], or dCas9‐sRNA complex) can by themselves obstruct the access of RNA polymerase, resulting in gene knockdown, but coupling with KRAB domains further enhances transcriptional inhibition.^[^ ^188^ ^]^ Recently, a screening of 57 human KRAB domains was conducted, and among them, the KRAB from the ZIM3 gene was found to be the most potent in dCas9‐based targeted gene repression.^[^ ^189^ ^]^ Nevertheless, KRAB‐based systems suffer from inefficient knockdown, and combination with other repression domains that directly catalyze epigenome modifications results in improved performance.^[^ ^190^, ^191^ ^]^

DNA is wrapped around histone proteins (H2A, H2B, H3, and H4) forming the basic units of chromatin, the nucleosomes. Chemical modifications of histones, such as acetylation and methylation can affect the structure of chromatin and the accessibility of DNA to transcriptional machinery.^[^ ^192^, ^193^ ^]^ Acetylation involves the addition of acetyl groups to lysine residues (for instance, K9 and K27 of histone H3), which neutralizes the positive charge and weakens the interaction between histones and DNA. As a result, the chromatin structure becomes more accessible to transcriptional machinery, allowing for increased gene expression.^[^ ^194^ ^]^ Histone methylation can be linked to either activation or repression, depending on the specific amino acids and the degree of methylation. For instance, tri‐methylation of H3K9 is a key marker of transcriptional silencing^[^ ^195^ ^]^ while tri‐methylation of H3K4 is associated with gene activation.^[^ ^196^ ^]^ DNA methylation involves the addition of a methyl group to the cytosine base in CpG dinucleotides, which typically represses gene expression, as it can block the binding of transcription factors and attract proteins that silence gene activity.^[^ ^197^ ^]^ Examples of chromatin‐modifying enzymes include histone deacetylases (HDACs), methyltransferases, and acetyltransferases.^[^ ^198^ ^]^ Some of these enzymes have been coupled with ZF and TALE DBDs or clustered regularly interspaced short palindromic repeats (CRISPR)/dCas9 to artificially modify epigenetic marks on specific genes, thereby enabling the targeted activation or silencing of their expression.^[^ ^199^, ^200^, ^201^ ^]^ These approaches enable targeted modulation of the epigenetic landscape at specific gene loci.

### 4.1 Small‐Molecule–Inducible Systems

The tetracycline transrepressor TetR has been by far the most commonly employed bacterial TF to regulate gene expression in mammalian cells. In its first application, it was fused to the herpes simplex virus transactivation domain VP16.^[^ ^59^ ^]^ Binding of tetracycline or its derivatives (e.g., doxycycline) to homodimerized TetR‐VP16 causes a conformational change that leads to its dissociation from Tet operator DNA elements, precluding expression of the downstream gene (Figure 3b). This is an OFF system, since the addition of the inducer shuts off gene expression. To create an ON system, TetR was mutated in four residues which reversed its DNA binding behavior.^[^ ^71^ ^]^ This variant, known as rTetR, requires tetracycline for binding to the Tet operator sites, thereby driving target gene expression when fused to activation domains. Similar to TetR, the transrepressor VanR, which controls the vanillic acid (VA) utilization pathway in the gram‐negative bacterium *Caulobacter crescentus*, has been engineered for use in mammalian gene switches, both as OFF‐type^[^ ^60^ ^]^ and ON‐type systems,^[^ ^24^, ^60^ ^]^ in response to the plant‐derived inducer molecule VA. This system has been optimized for use in induced pluripotent stem cells, to control their differentiation toward desired cell lineages.^[^ ^72^, ^73^ ^]^

More recent systems employ metabolites from bacterial central carbon metabolism.^[^ ^6^, ^7^, ^9^ ^]^ In glucose‐limiting conditions, *Escherichia coli* (*E. coli*) can utilize alternative carbon sources, including xylose and gluconate. The orchestration of protein synthesis required for metabolizing these compounds relies on the transcription factors XylR or GntR, each binding to specific DNA sequences in a metabolite‐dependent manner. XylR forms antiparallel dimers upon xylose binding, triggering structural alterations that facilitate its interaction with DNA operator sites.^[^ ^74^ ^]^ The mechanism of GntR binding to DNA is less well studied, but the minimal operator sites to which it binds have been identified.^[^ ^75^ ^]^ Leveraging these molecular elements, we have recently introduced two new orthogonal gene switches that offer tight control in response to xylose or gluconate.^[^ ^6^, ^7^ ^]^ The expression of heterologous transporters for each metabolite enhanced their uptake by mammalian cells, establishing highly sensitive gene switches that respond effectively in the lower micromolar range (Figure 3b). The practical application of these gene switches for regulation of transgene expression was demonstrated in mouse studies.

### 4.2 Light‐Inducible Systems

The light‐regulated DNA‐binding protein EL222 from *Erythrobacter litoralis* contains an HTH DNA‐binding domain and a photosensory LOV domain, which harness internally bound flavin chromophores to detect variations in light conditions. In the dark, the LOV and HTH domains interact, preventing DNA‐binding, while under blue‐light illumination this interaction is disturbed, making the protein DNA‐binding competent.^[^ ^76^ ^]^ This photosensitive protein has been employed to build single‐protein light‐inducible gene switches in mammalian cells.^[^ ^69^ ^]^ Fusion to a mammalian activation domain allowed blue‐light–inducible reporter gene expression from a promoter containing optimized EL22 binding sites, reaching over 100‐fold induction relative to cells kept in the dark. Notably, this system exhibits high sensitivity to relatively low‐intensity blue light, triggering prompt gene expression upon illumination and rapid deactivation once the dark state is restored. Another transactivator system was achieved with Vivid, a LOV‐containing protein from *Neurospora crassa*. The short DNA‐binding domain of Gal4, consisting of the first 65 residues at the *N*‐terminus lacks the ability to dimerize and, consequently, cannot bind to its cognate DNA sequence (UAS). To develop a light‐switchable gene expression system, Wang et al.^[^ ^77^ ^]^ fused this Gal4 domain with Vivid and the p65 transactivation domain. Upon activation with blue light, the LOV domain undergoes homodimerization, thus facilitating the binding of Gal4 to its promoter region, and initiating the expression of the desired transgene. Both systems have a reduced genetic footprint compared with alternative multicomponent light‐controlled gene expression methods^[^ ^78^, ^79^, ^80^ ^]^ and use the natural co‐factor FAD as a photon acceptor, thus avoiding the need to treat cells with external chromophores.

### 4.3 Temperature‐Inducible Systems

The human pathogen *Salmonella typhimurium* encodes the temperature‐sensing transcription factor TlpA, which contains a *C*‐terminal α‐helical coiled‐coil motif and a sequence‐specific DNA‐binding domain. At cooler environmental temperatures, TlpA forms a dimeric folded coiled‐coil conformation that can bind to and repress the tlpA promoter. At warmer intracellular temperatures around 37 °C, TlpA adopts a non‐functional unfolded monomeric form which cannot bind DNA. Capitalizing on a variant which activates at higher transition temperatures (TlpA_39_^[^ ^68^ ^]^), our group established a temperature‐sensitive gene switch in mammalian cells. We found that a tetracycline‐dependent transactivator (tTA) fused *C*‐terminally to TlpA_39_ is locked in an inactive homodimerized configuration by the TlpA coiled‐coil domains at 37 °C. At higher temperatures (40 °C), the TlpA moiety adopts an unfolded monomeric state, releasing functional tTA, which can bind to and activate tetracycline‐responsive promoters.^[^ ^19^ ^]^

These examples demonstrate the broad adaptability of microbial TFs to build mammalian gene switches that specifically respond to different chemical or physical environmental changes. Well‐characterized TFs with predicted specificities for DNA, and that do not require additional factors from their native hosts, can be readily adapted for use in mammalian cells. To be able to use TFs that are responsive to molecules of interest but with unknown operator sites, we recently developed a framework based on chimeric TFs.^[^ ^24^ ^]^ We have shown that some bacterial TFs (including those responsive to acetoin, xylose, gluconate, among others), also exhibit responsiveness to their effectors when fused to another TF, relying on the interaction of the latter TF with its cognate DNA‐binding sequence for the regulation of gene expression. For instance, by fusing GntR to TetR and to a transactivation domain (e.g., VP16), gluconate‐inducible gene expression is obtained from a tetracycline‐responsive promoter as GntR dimerization brings VP16 near the target promoter.^[^ ^6^, ^24^ ^]^ This framework allowed us to establish high‐performance gene switches responsive to new inducers, which were integrated in large logic gates of up to five inputs.

This is by far the largest category of macromolecules that can be regulated by both a diverse library of small molecules and by physical signals. Notwithstanding, bacterial TFs are foreign proteins in mammalian cells, and thus may trigger immune responses or show unintended binding to off‐target DNA sequences, potentially causing undesired gene expression changes. Extensive characterization and validation of synthetic gene switches are necessary to ensure specificity and minimize off‐target effects. Another inherent issue with gene switches based on bacterial TFs is their dependence on specific DNA sequences for binding, which limits their scope primarily to the regulation of exogenously delivered transgenes. Nevertheless, despite the complexities of inserting operator sites in the promoter region of endogenous genes, this has been achieved. For instance, LacO sequences were introduced at selected sites in the Dnmt1 promoter by homologous recombination, and insertion sites that minimally affected endogenous gene regulation were identified.^[^ ^81^ ^]^ Alternative strategies to minimize potential limitations associated with bacterial components include the use of human transcription factors. For instance, cells overexpressing the components of the antioxidative pathway KEAP1/NRF2 become sensitive to reactive oxidative species generated by exposure to direct current and activate transgene expression from a highly optimized promoter containing antioxidant‐responsive elements.^[^ ^18^ ^]^ Greater versatility can be achieved with artificial DBDs based on zinc‐finger (ZF) motifs, TALE proteins, or CRISPR‐dCas systems (Box 2) fused to mammalian‐specific transcription factors. These tools are particularly well‐suited for endogenous gene regulation, since unlike natural bacterial or human transcription factors, they do not require the introduction of the DNA‐binding sites into the cell genome.

**Box 2. Tools for Precise Gene Regulation and Genome‐Editing**

Artificial zinc‐fingers (ZFs) are customized DBD containing multiple zinc‐finger motifs, each composed of 28 residues that recognize and bind to a specific DNA triplet.^[^ ^202^, ^203^ ^]^ By assembling six to eight zinc‐finger domains in a specific order, longer DNA sequences (18 to 24 bp) can be targeted with high specificity. Zinc‐finger transcription factors are obtained by attaching transcription activation or repression domains to the ZF effector domain. Conversely, ZF nucleases (ZFNs) are obtained by fusing the catalytic domain of the type IIS restriction endonuclease FokI.^[^ ^204^ ^]^ Because FokI is catalytically active as a dimer, a pair of ZFN subunits is assembled at the target site, leading to the simultaneous cleavage of both DNA strands within the spacer between the two ZF target sites, generating two 5′‐overhang DNA ends. Each ZFN subunit typically consists of three to four zinc‐finger domains that together recognize 9 to 12 bp, respectively. Careful design and validation of zinc‐finger proteins is required to avoid off‐target effects in gene regulation/editing. Computational approaches can be used to simplify the design process.^[^ ^205^ ^]^

Artificial TALEs are customized DNA‐binding domains, consisting of a series of tandem repeats, typically 33–35 amino acids long, where each repeat targets a single DNA base pair.^[^ ^206^ ^]^ The specificity of the DNA binding is determined by two variable amino acid residues within each repeat (typically at positions 12 and 13), known as the repeat variable diresidues (RVDs). TALEs are natural proteins from *Xanthomonas* bacteria, which are plant pathogens. The four most common RVDs are His–Asp for cytosine, Asn–Iso for adenine, Asp–Gly for thymine, and Asn–Asn for guanine.^[^ ^207^ ^]^ Compared to ZFs, the process of programming TALEs to target new sequences is more straightforward. Like ZFNs, TALE nucleases (TALENs) are obtained by attaching the nuclease domain of FokI. TALENs are designed to bind a DNA target, usually consisting of two sequences of 16 nucleotides separated by a spacer sequence of 15–16 nucleotides where the cleavage occurs. TALEs have also been successfully customized for endogenous gene regulation in human cells, using transcription activation or repression domains as effectors.^[^ ^208^ ^]^

The CRISPR system, adapted from a microbial defense mechanism, combines a CRISPR‐associated (Cas) endonuclease and a chimeric RNA called single guide RNA (sgRNA) that contains a 20–30 nucleotide sequence complementary to the target DNA sequence.^[^ ^209^ ^]^ While the specificity of ZFs and TALEs is dependent on protein–DNA interactions, the specificity of the CRISPR system is achieved through the base‐pairing between the sgRNA and the target DNA sequence. The presence of the protospacer adjacent motif (PAM) immediately downstream of the target site in the DNA is crucial for Cas protein recognition and binding. The CRISPR‐Cas9 system derived from *Streptococcus pyogenes* is the most widely used. SpCas9 cuts both DNA strands a few bp upstream of the PAM sequence (5′‐NGG‐3′, where N can be any nucleotide), generating blunt ends, as opposed to artificial ZFNs and TALENs which produce overhangs. In contrast to the complex protein engineering associated with ZFs and TALEs, the CRISPR‐Cas system is an extremely flexible tool that can be easily guided to target almost any desired location in the genome by designing an appropriate gRNA. Beyond genome editing, CRISPR has been repurposed to achieve targeted gene regulation or epigenome editing. Two mutations render dCas9, thus abrogating its ability to cleave DNA but retaining the ability to be guided to the target sequence.^[^ ^209^ ^]^

There are two primary repair pathways for DSBs introduced by the nucleases described above: non‐homologous end joining (NHEJ) and homology‐directed repair (HDR).^[^ ^210^ ^]^ NHEJ is an error‐prone repair mechanism that often leads to small insertions or deletions at the cleavage site. On the other hand, HDR uses a homologous DNA template to repair the DSB with high precision. Introducing the synthetic DNA template along with the nuclease, the HDR pathway can use this as a blueprint to precisely repair the DNA at the DSB site. This repair process can be harnessed to achieve targeted genome modifications, such as gene insertion, correction of mutations, or gene knockout.

## 5 Transcription Regulation Relying on Programmable DNA‐Binding Domains

**Figure 4:**
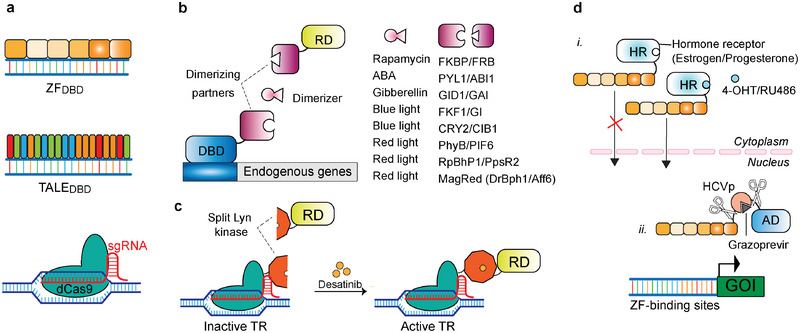
Designer transcription factors for regulation of transgenes and endogenous genes in mammalian cells. a) Designer DNA‐binding domains (DBD) consist of artificial zinc‐fingers (ZF), transcription activator‐like effectors (TALE) or small guide RNA (sgRNA)‐directed dCas9 protein (Box 2). b) Two‐component designer transcriptional regulators are assembled by fusing the designer DBD and the regulatory domain (RD) to the two complementary partners of chemically induced or light‐induced dimerization systems. c) Designer transcriptional regulators (TR) using split human ligand‐binding proteins. In the example shown, the split fragments of Lyn kinase fused to dCas9 or a RD assemble a TR in the presence of the small‐molecular drug desatinib. d) Single‐chain designer TRs. i. Variants of the estrogen or progesterone hormone receptors (HR) fused to designer ZF_DBD_ are sequestered in the cytoplasm by default but can translocate to the nucleus in the presence of the synthetic ligands 4‐OHT or RU486, respectively. ii. Alternatively, the DBD and an activation domain (AD) can be fused in a single‐chain protein with an inhibitable protease and a protease cleavage sequence between them. In the example shown, the hepatitis C virus protease (HCVp) cleaves the designer TR unless its inhibitor grazoprevir is present, so that the inhibitor allows transcription of the downstream GOI to occur.

Designer transcription regulators consist of a customizable DNA‐binding module based on ZFs, TALEs, or sgRNA/dCas9 (**Figure** **4**a), and a transcription effector module.^[^ ^82^, ^83^, ^84^, ^85^ ^]^ For endogenous gene regulation, the DBDs are designed to recognize specific DNA sequences in the promoter region of the target genes. Multiple DBDs targeting different sequences in the same region are often screened to identify the best‐performing domains, considering factors such as specificity and absence of off‐target effects. While engineering ZFs or TALEs requires constructing custom protein domains for each target sequence, which makes the process time‐consuming and complex, CRISPR‐Cas systems utilize an easily designed and synthesized short guide RNA (sgRNA) sequence for targeting the desired DNA sequence (Box 2). Therefore, CRISPR‐Cas systems exhibit higher scalability and can be easily multiplexed to target multiple genomic loci simultaneously, thus allowing complex genetic manipulations that would be more challenging with ZFs or TALEs. However, single‐gene targeting with ZFs allows for more compact TFs, and the widespread presence of natural ZFs in the human proteome may render artificial ZFs less immunogenic compared to artificial TALEs and CRISPR‐Cas tools, which naturally originate from bacteria.

### 5.1 Assembling Designer Transcription Regulators Using Small Molecules

To achieve conditional activation of ZF‐, TALE‐, or dCas9‐based transcription factors or epigenome modifiers, enabling artificial regulation of endogenous genes in response to environmental signals, a modular approach involves coupling them with protein domains that dimerize upon detection of the chosen signal (Figure 4b). In such two‐component systems, a full transcription regulator is reconstituted from two split parts: the DBD and the activation/repression module (Box 1) are fused to complementary pairs of dimerizing domains. When the dimerization signal is supplied, the transcription effector is recruited to the target promoter region, resulting in the activation/repression of gene expression.

#### 5.1.1 Dimerization Using “Rapalogs”

The macrolide rapamycin is the prototypical example of a chemical dimerizer. Rapamycin binds to the pocket of FKBP12, the smallest member of the FK506‐binding protein (FKBP) family, present in all eukaryotes. The rapamycin‐FKBP12 complex can bind to the FKBP rapamycin binding (FRB) domain of the mammalian target of rapamycin (mTOR), inhibiting its kinase activity. The fundamental concept underlying chemically‐induced dimerization (CID) using rapamycin or its analogs (rapalogs) is based on tethering two proteins of interest (POIs) to the complementary pairs of heterodimerization domains (FKBP and FRB) to induce interaction between the POIs by addition of rapamycin.^[^ ^86^ ^]^ To avoid the immunosuppressive properties of rapamycin and the unspecific binding to endogenous mTOR, rapamycin derivatives that can only bind specific FRB mutants have been developed.^[^ ^23^, ^87^ ^]^ This approach has been used to control various cellular processes, including the activation of receptors^[^ ^88^ ^]^ and the regulation of exogenous or endogenous genes (e.g., refs. [24, 86, 89]).

Some studies have employed the rapamycin‐inducible heterodimerizing domains to reconstitute split fragments of dCas9 for transcriptional regulation.^[^ ^90^ ^]^ When the two fragments are brought together by the chemical input, they can reconstitute fully functional dCas9 proteins. However, the reported background activity in the absence of the dimerizer remained relatively high, even when one of the dCas9 fragments was localized to the cytosol.

Interestingly, chemically induced trimerization was made possible recently with a derivation of this system. Either the FKBP or FRB domain is split into two fragments, and the three fragments can be reassembled in the presence of rapalogs, thereby promoting the interaction of three proteins of interest.^[^ ^91^ ^]^ This advance paves the way for the construction of increasingly intricate synthetic circuits to engineer unique cellular behaviors and the execution of sophisticated logic computations within human cells.

Furthermore, heterobifunctional molecules formed by covalently linking a ligand that binds to the FKBP(F36V) variant with different ligands of the cellular chromatin‐modifying machinery have been used to associate dCas9‐FKBP(F36V) with endogenous chromatin‐modifiers.^[^ ^92^, ^93^ ^]^ This strategy was effective to deposit active marks at different genomic loci to modulate endogenous gene expression in a specific, dose‐dependent manner, using synthetic proximity‐inducing ligands.

#### 5.1.2 Dimerization Using Plant Hormones

Other chemically inducible dimerization domains are based on plant‐derived abscisic acid (ABA) or gibberellin. The phytohormone ABA protects plants from environmental stresses. In *Arabidopsis thaliana* (*A. thaliana*), ABA binds to the PYL1 receptor, and the resulting complex associates with the negative regulator ABI1, inhibiting its phosphatase activity.^[^ ^94^ ^]^ Based on the crystal structure of the ternary complex PYL1‐ABA‐ABI1, Liang et al.^[^ ^95^ ^]^ devised truncations of both proteins comprising their interacting complementary surfaces and applied them to induce proximity between different pairs of proteins in an ABA‐responsive manner. In the absence of ABA, the PYL receptor cannot interact with ABI due to masking of the interaction domain. Similarly, gibberellins are plant hormones which regulate various aspects of plant growth and development. When gibberellins bind the receptor gibberellin insensitive dwarf1 (GID1), the protein undergoes conformational changes, allowing for the recruitment of and interaction with another protein called gibberellin insensitive (GAI).^[^ ^96^ ^]^ Miyamoto et al.^[^ ^96^ ^]^ screened various truncations of GID1 and GAI to achieve the minimal domains that can form a CID system activated by GA_3_‐AM, a gibberellin metabolite which readily enters mammalian cells and is cleaved by esterases to release active GA_3_.

These systems have been applied to bring KRAB domains into proximity with target promoters for gene silencing, as they recruit epigenetic modifiers of histone marks and DNA methylation that condense the chromatin. When fusing dCas9 and KRAB with the respective complementary partners of the ABA‐ and gibberellin‐inducible heterodimerizing domains, repression of genomically encoded fluorescent reporters was obtained in response to the plant hormones.^[^ ^97^ ^]^ In another application, the catalytic core of the histone acetyltransferase P300 was recruited to deposit H3K27ac at target endogenous promoters, relying on the ABA heterodimerization system to promote dCas9 and P300 interaction.^[^ ^98^ ^]^ This study achieved up to 30‐fold increase in the mRNA levels of target genes in cells treated with the inducer, relative to non‐treated cells.

#### 5.1.3 Dimerization Using Novel Split Human Proteins and Their Ligands

A recent study has considerably enriched the toolkit of CID systems, all based on split human ligand‐binding proteins.^[^ ^99^ ^]^ This work involved the identification of split sites within six distinct proteins, such that the two generated fragments could be brought together when exposed to the corresponding ligands (either natural ligands or synthetic agonist/antagonist molecules). The split proteins that can function as new CIDs (and the most potent ligands found to induce the respective reassembly of the two fragments) are Lyn kinase (desatinib), DHFR enzyme (methotrexate), and four nuclear receptors: glucocorticoid receptor 2 (mometasone furoate), peroxisome proliferator‐activated receptor gamma (rosiglitazone), thyroid receptor beta (hormone triiodothyronine), and estrogen receptor beta (4‐hydroxytamoxifen, 4‐OHT). These CID systems were successfully applied to reconstitute TALE‐ and dCas9‐based regulators to control the expression of transgenes and endogenous genes (Figure 4c). Nonetheless, due to the origin of these systems in naturally occurring human proteins, the small molecules employed are likely to have off‐target effects. Evolution of these CIDs alongside their corresponding ligands to minimize any potential interference with endogenous proteins should unlock their full potential for medical applications.

### 5.2 Designer Transcription Regulators Responsive to Synthetic Hormone Analogues

Chemogenetic approaches based on the LBD of nuclear estrogen and progesterone receptors (ER and PR) have been developed, relying on their ability to dissociate from inactivating complexes in the cytoplasm and translocate to the nucleus in the presence of the corresponding hormones. Variants of ER and PR LBDs that no longer respond to natural ligands but remain sensitive to the synthetic ligands 4‐OHT or RU486 have been combined with ZFs^[^ ^84^, ^100^ ^]^ and TALEs^[^ ^101^ ^]^ fused to activation domains to develop single‐chain transactivators that are tunable in a small‐molecule–dependent manner (Figure 4d). Li et al.^[^ ^84^ ^]^ created orthogonal ZF transcription factors based on the ER LBD and the two‐component ABA‐inducible system. Both systems could regulate reporter transgenes in Jurkat cells with minimal basal expression and wide dynamic ranges. However, the 4‐OHT–regulated system exhibited a 20‐fold higher fold‐induction compared to the ABA‐inducible system, prompting its adoption for the regulation of therapeutic payloads in primary human T cells and in vivo regulation of CAR‐T cells.^[^ ^84^ ^]^

### 5.3 Assembling Designer Transcription Regulators Using Light

Several light‐inducible dimerization (LID) systems have been applied to drive the association between a DBD and an effector domain for transcriptional regulation. By fusing light‐sensitive domains, such as LOV or phytochrome domains to proteins of interest, we can control their association by irradiation with specific wavelengths of light.

#### 5.3.1 Blue‐Light–Inducible Dimerization Systems

The interacting partners from two blue‐light–inducible dimerization systems derived from the plant *A. thaliana* (FKF1/GI and CRY2/CIB1) have been fused to DBDs based on either ZFs^[^ ^78^ ^]^ or TALEs^[^ ^102^ ^]^ and to a regulatory effector (VP16, VP64, or epigenome modifiers). This enables bringing the effector domain into close proximity with the DBD bound to its target DNA sequence upon illumination, thereby modulating gene expression.^[^ ^78^, ^102^ ^]^ The CRY2/CIB1‐based system has also been used for photoactivation of dCas9‐based transcription.^[^ ^103^ ^]^ The gRNAs direct the binding of dCas9‐CIB1 to the promoter of the target gene and CRY2‐VP64 colocalizes with dCas9 via CRY2‐CIB1 interactions upon exposure to blue light, consequently inducing transcription. Conversely, in the absence of blue light, the domains remain in their monomeric state, preventing transcriptional changes.

One of the key distinctions between these blue‐light–triggered systems lies in the nature of their interactions. Upon light stimulation, FKF1 undergoes a non‐reversible interaction with GI, whereas the interaction between CRY2/CIB1 is reversible. This difference is crucial when choosing between the two systems. For example, the FKF1/GI system does not need continuous exposure of live cells to light, potentially reducing the risk of phototoxic effects. Both systems have been optimized by Quejada et al.,^[^ ^104^ ^]^ and a direct comparison revealed that the FKF1/GI‐based system exhibited a lower background signal under dark conditions.

Nihongaki et al.,^[^ ^105^ ^]^ achieved superior performance with a photoactivatable split dCas9 approach. The two split dCas9 fragments were fused to blue‐light–inducible LOV domains known as positive (pMag) and negative (nMag) magnets, and one of the fragments was flanked by nuclear export signals to keep it outside of the nucleus. Upon blue‐light exposure, the two assembled dCas9 fragments localize to the target promoter and, together with the synergistic action of three activation domains (VP64, p65, and HSF1), significantly increase the magnitude of the upregulation achieved with previous photoactivable dCas9 systems.^[^ ^103^, ^105^ ^]^ Nevertheless, some background activity was still observed which might not be tolerable for high‐precision regulation requirements. For such cases, photoactivatable (non‐split) dCas9‐based transcription factors might be better suited.

#### 5.3.2 Red‐Light–Inducible Dimerization Systems

Owing to the increased tissue penetration of light in the red wavelength region, considerable research efforts have been directed to the discovery and application of LID systems responsive to red or near‐infrared light. In this context, the red‐light–induced interactions of phytochrome B and phytochrome interacting factor 6 (PIF6) from *A. thaliana*,^[^ ^79^ ^]^ and the bacteriophytochromes RpBphP1 and PpsR2 from *Rhodopseudomonas palustris*, which interact upon near‐infrared (NIR) irradiation, have been employed to assemble split transactivators based on TetR and VP16 upon exposure to light.^[^ ^80^ ^]^ Although both systems achieved similar fold‐inductions in reporter gene expression from a TetR‐dependent promoter, the NIR‐based system is preferable for in vivo applications, due to its capacity for deeper tissue penetration, coupled with its reliance on a chromophore (biliverdin) that is available endogenously in mammalian cells, as opposed to the plant‐derived system. Nevertheless, a high level of leaky expression in the dark was associated with this system in recent dCas9‐based photoactivable gene expression experiments.^[^ ^106^ ^]^ MagRed is a novel red‐light–inducible dimerization method for direct manipulation of protein activity that solves this issue.^[^ ^106^ ^]^ It consists of a red‐light‐responsive phytochrome derived from *Deinococcus radiodurants* (DrBphP) and a photo‐state–specific synthetic partner developed using Affibody library selection. MagRed enabled the recruitment of transactivation domains in response to red light exposure to regulate endogenous genes based on dCas9, resulting in high fold‐inductions from multiple genomic loci, while minimizing leaky activity in the dark. Furthermore, the two protein domains fused to the MagRed components can be repeatedly assembled and disassembled by pulsed light at different wavelengths (660 and 800 nm, respectively).

These dimerization systems have facilitated precise control over protein–protein interactions of split transcriptional effectors used for regulating exogenous or endogenous genes in mammalian cells. By utilizing small molecules or light illumination to regulate the interaction between specific protein domains, these systems enable spatiotemporal control over transcriptional effector activity. Six of the outlined chemical‐ or light‐inducible dimerization systems were compared side‐by‐side to develop dCas9‐based transcription regulation systems, aiming at associating dCas9 with VPR at the promoter of a reporter gene.^[^ ^97^ ^]^ The ABA and gibberellin heterodimerization systems exhibited superior performance relative to the classical rapamycin system, as well as the blue‐light–inducible FKF1/GI and CRY2/CIB systems and the red‐light–inducible PHYB/PIF system. However, the choice of system ultimately depends on the specific application at hand.

## 6 Synthetic Devices Relying on Translation Regulation

The broad spectrum of naturally inducible DNA‐binding proteins, complemented by the flexibility to engineer novel DNA‐binding domains tailored to specific promoter sequences, and their modularity to be integrated with transcription effector domains into larger systems for advanced functionalities, collectively support the widespread adoption of systems regulated at the transcriptional level. However, synthetic circuits relying solely on transcriptional regulation are associated with high metabolic loads and prolonged response times due to the necessity of processing every step of gene expression. Conversely, translationally regulated systems offer faster dynamics by circumventing the transcriptional phase, operating with greater efficiency in terms of nutrient requirements, and imposing less stress on the cellular protein synthesis machinery.

**Figure 5:**
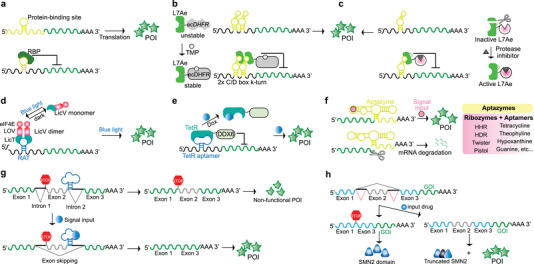
RNA‐based switches for translational regulation of transgenes. a) RNA‐binding proteins (RBP), such as L7Ae, bind to RNA structures placed in the 5′ UTR of the GOI, thereby preventing translation initiation. b–e) External regulation of RBPs. b) RBPs can be fused to destabilizing domains, such as the DHFR variant from *E. coli*, to enable the regulation of their degradation by the small molecule TMP, or they can be fused to c) an inhibitable protease that acts on an inserted protease cleavage site in an exposed loop of the RBP, to enable the regulation of their abundance in response to a protease inhibitor. d) Photoswitchable RBP. The fusion of the transcriptional antiterminator protein LicT from *B. subtilis* to a LOV domain‐containing protein that dimerizes upon exposure to blue light facilitates its binding to a ribonucleic antiterminator (RAT) RNA sequence, preventing the formation of an RNA terminator stem–loop structure under blue‐light illumination. The resulting protein LicV fused to translation initiation factor 4E (eIF4E) directs the translation machinery to the 5′ UTR of the target mRNA and initiates translation. In the dark, the fusion protein dissociates from the RNA, preventing translation. e) Dox‐regulated translation. The TetR protein fused to mammalian dead box helicase 6 (DDX6) protein binds to its RNA aptamer placed in the 5′ UTR of a GOI in the absence of Dox, thereby repressing translation, which is relieved when Dox is introduced. f) Aptazyme‐based riboswitches. Self‐cleaving ribozymes (e.g., HHR, HDR, Twister, or Pistol) can be allosterically controlled by incorporating small‐molecule–responsive aptamers (e.g., tetracycline, theophylline, hypoxanthine, guanine, etc.) in their structures. Placing the resulting aptazymes into the 3′‐UTR of a target gene allows regulation of the transcript levels. In the example shown, the small molecule prevents the removal of the poly‐A tail via ribozyme‐mediated self‐cleavage, stabilizing the transcript and thereby increasing the mRNA levels in a concentration‐dependent manner. g,h) Exon‐skipping riboswitches. g) A stop codon‐containing exon (exon 2) flanked by introns 1 and 2 is inserted between two exons of the GOI (exon 1 and 3). A ligand‐responsive aptamer is inserted downstream of the 5′ splicing sequence of intron 2. In the absence of the ligand, exon 2 is incorporated into the mature mRNA, thereby resulting in non‐functional POI. In the presence of the ligand, the splicing sequence is masked by the aptamer structure, leading to exon 2 skipping and thereby allowing expression of functional POI. h) Three exons from the SMN2 gene, featuring the middle exon flanked by two intronic sequences, are placed upstream of a GOI. When a SMN2 splicing‐regulating small drug is present, the GOI is in‐frame and is actively expressed. Conversely, in the absence of drug treatment, a stop codon is present that prevents the translation of the GOI.

Synthetic RNA‐based switches embedded in the target transcript can be designed to respond to different signals, including proteins, small molecules, and oligonucleotides. Translational control in response to protein stimuli can be achieved by employing protein‐responsive RNA‐based switches within the 5′‐untranslated region (UTR) of mammalian transcripts.^[^ ^107^ ^]^ This strategy capitalizes on RNA‐binding proteins (RBPs) coupled with their cognate RNA‐binding motifs, such as the archaeal L7ae protein, which engages with the RNA C/D box k‐turn, and the bacteriophage MCP, which binds MS2 RNA stem‐loops (**Figure** **5**a). The assembled ribonucleoprotein complexes impose their regulatory influence by decreasing the translational efficiency through steric hindrance. One potential approach to achieve external control over these protein‐responsive RNA switches involves engineering the RBP with appropriate modules to regulate its abundance in a signal‐input–dependent manner. This can be achieved through the incorporation of inducible destabilizing domains (DD)^[^ ^108^ ^]^ (Figure 5b) or the integration of inhibitable proteases along with the corresponding cleavage sites (Figure 5c).^[^ ^8^, ^30^, ^31^, ^109^ ^]^ These manipulations equip the RBP to undergo controlled inactivation in response to the presence of specific small molecules. For instance, Wagner et al. fused a trimethoprim (TMP)‐responsive DD to L7Ae to decrease translation repression of mRNAs containing a K‐turn motif in the 5′‐UTR due to DD‐mediated degradation.^[^ ^108^ ^]^ The addition of TMP stabilizes the fusion protein DD‐L7Ae, which can bind to the target RNA, thereby repressing translation of the target genes. Manipulation of RNA dynamics has also been achieved using light as a trigger. A photoswitchable RBP termed LicV was engineered by fusing two bacterial proteins: i) the RNA‐binding domain of the *Bacillus subtilis* (*B. subtilis*) LicT protein, which binds as a dimer to a specific RNA sequence to prevent the formation of an RNA terminator stem–loop structure and ii) the LOV‐containing protein Vivid, which dimerizes following activation with blue light (Figure 5d).^[^ ^110^ ^]^ For optogenetic control of mRNA translation in mammalian cells, the translation initiation factor 4E (eIF4E) was attached to LicV and upregulation of target genes bearing multiple recognition RNA binding sites in the 5′‐UTR was observed upon exposure to blue light.

Direct ligand‐induced interaction between a RBP and its cognate RNA sequence for post‐transcriptional control has been accomplished using doxycycline (Dox) and TetR aptamers. RNA‐binding motifs with high affinity and specificity for the TetR protein were identified using systematic evolution of ligands by exponential enrichment (SELEX) technology.^[^ ^111^, ^112^ ^]^ Integrating a TetR aptamer into the 5′‐UTR of target mRNAs leads to decreased translation efficiency at baseline when the TetR protein is co‐expressed (Figure 5e). The suppression of translation is directly alleviated by Dox treatment, which disrupts the interaction between TetR and the RNA aptamer. Translational repression by doxycycline was improved when TetR was fused to dead box helicase 6 (DDX6), an evolutionarily conserved protein that interacts with mRNA degradation proteins.^[^ ^108^ ^]^ Translational repression has been also achieved through direct light‐inducible protein–RNA interactions.^[^ ^113^ ^]^ This strategy capitalizes on the bacterial blue‐light LOV receptor PAL, which, based on homology, was hypothesized to possess RNA‐binding properties despite an elusive binding sequence. RNA aptamers showcasing light‐responsive affinity to PAL were isolated using SELEX. By incorporating the PAL aptamer within the 5′‐UTR of a luciferase protein, translational repression was achieved in mammalian cells co‐expressing PAL, upon exposure to blue light.

However, despite being faster‐acting than transcriptionally based switches, the outlined post‐transcriptional regulatory switches still depend on accessory regulatory proteins which impose some limitations, namely the need to co‐introduce expression constructs for both the RBP and the transgene to be regulated and the potential toxicities due to expression of a foreign protein.

A different class of post‐transcriptional regulatory switches relies on self‐cleaving ribozymes, which are RNA sequences capable of catalytic activity. Introducing cis‐acting ribozymes into the 3′‐UTR of a target gene causes the removal of the poly‐A tail via ribozyme‐mediated self‐cleavage, thereby promoting rapid degradation of the transcript.^[^ ^114^ ^]^ Allosteric control of ribozymes can be achieved by incorporating aptamers in their structures (Figure 5f). The aptamer replaces a stem loop within the ribozyme, and by optimizing the communication linker between these elements, the catalytic activity of the ribozyme becomes amenable to regulation by the small‐molecule ligand. The binding of the ligands to the aptamers induces conformational changes that directly influence the catalytic activity. In on‐type riboswitches, the act of ligand binding to the aptamer domain inhibits ribozyme self‐cleavage, stabilizing the transcript, and thereby increasing the mRNA and protein levels in a concentration‐dependent manner.

Various self‐cleaving ribozymes have been used as scaffolds to engineer mammalian riboswitches, namely hammerhead,^[^ ^27^, ^28^, ^29^, ^114^, ^115^ ^]^ hepatitis delta virus,^[^ ^116^ ^]^ twister ribozymes, and synthetic variants of pistol ribozymes.^[^ ^117^ ^]^ Adapting ribozyme‐aptamer riboswitches (termed aptazymes) to exhibit efficient switching behavior in mammalian cells involves extensive screening, for instance to optimize the linkers that connect the ribozymes to the aptamers. The RNA aptamers obtained by in vitro selection methods do not always function in vivo, and even those functioning in bacteria or lower eukaryotes still need extensive in vivo screening in mammalian cells before a riboswitch with satisfactory regulatory performance can be obtained. The tetracycline and theophylline aptamers discovered by SELEX and the bacterially derived guanine aptamer have been the most widely explored to develop improved mammalian riboswitches.^[^ ^28^, ^115^, ^118^, ^119^ ^]^

In an initial study in mammalian cells, synthetic‐drug–responsive, hammerhead ribozyme‐based RNA devices were applied to regulate cytokine levels in mouse T cells, thereby controlling cell proliferation.^[^ ^28^ ^]^ Theophylline‐ and tetracycline‐inducible riboswitches placed in the 3′ UTR of cytokine transgenes undergo ribozyme self‐cleavage, resulting in rapid degradation of the target transcripts and decreased cytokine production. Upon exposure to the molecular inputs, the ribozyme is inactivated, thereby preserving the transcript intact and up‐regulating cytokine production, resulting in cell proliferation. A similar tetracycline‐dependent riboswitch was developed by inserting a tetracycline aptamer into the hammerhead ribozyme in such a way that ligand binding to the aptamer destroys a loop–loop interaction within the ribozyme, thereby inhibiting ribozyme cleavage and allowing up to 8.7‐fold induction in Hela cells.^[^ ^115^ ^]^ Interestingly, aptazymes operating as off‐type switches, in which ligand binding to the aptamer activates the ribozyme, have been more successfully optimized in mammalian cells, achieving up to 30‐fold increases in reporter gene expression.^[^ ^11^, ^116^ ^]^ There are only a few aptazymes exhibiting the more desirable on‐type behavior, and these typically show lower fold‐changes (of up to 10).

Recently, Xiang et al.,^[^ ^120^ ^]^ introduced a high‐throughput methodology aimed at screening large libraries of synthetic ligand‐responsive aptazymes for robust functionality in mammalian cells. They were able to adapt ribozyme switches responsive to hypoxanthine, cyclic di‐GMP, and folinic acid, previously engineered in cell‐free or microbial contexts, to exhibit switching behavior in mammalian cells. Although the adapted switches exhibited low basal activity, their activation ratios were still limited to a maximum of ninefold. Additionally, cyclic di‐GMP and folinic acid are of limited utility in a mammalian cell context due to their poor cellular uptake. Overall, it has been challenging to bring the dynamic ranges closer to those achieved with transcription‐based switches, as well as to increase the set of ligand‐responsive riboswitches that are useful for human health applications.

The assembly of novel riboswitches capable of higher activation ratios in response to more favorable drug‐like compounds that are bioavailable and safe for human use would boost their applicability for cell and gene therapies. Indeed, the inherent small genetic footprint of usually less than 200 nucleotides of these RNA‐only devices makes them more attractive than inducible expression systems requiring co‐expression of heterologous regulatory proteins, such as transcription factors or RBPs, which are also potentially immunogenic. These features of synthetic aptazyme‐based riboswitches can be especially beneficial for gene delivery using the widely adopted adeno‐associated viral (AAV) vectors, which have limited packaging capacity. Numerous proof‐of‐concept mouse studies have demonstrated the functionality of aptazyme‐mediated regulation of AAV‐based transgene expression in vivo.^[^ ^11^, ^118^, ^121^, ^122^ ^]^

Engineering more efficient ribozymes may help to achieve wider dynamic ranges for modulation. In a recent study, Zhong et al.^[^ ^11^ ^]^ rationally designed new variants of the hammerhead ribozyme, exhibiting significantly enhanced self‐cleavage activity compared with the natural ribozyme (over 50‐fold greater, as measured in terms of the expression of a luciferase reporter protein in HEK cells). The optimized ribozyme enabled in vivo regulation of various AAV‐delivered reporter and therapeutic genes, when blocked with optimized antisense oligonucleotides, and dose‐dependent upregulation of protein expression by over 200‐fold was achieved. Furthermore, transgene expression could be repeatedly activated, and was still responsive to the steric oligos almost 1 year after AAV injection. However, the administration of oligo‐effectors for in vivo applications presents challenges, being less straightforward and convenient compared to small molecules. Combining the hyperactive ribozymes with RNA aptamers that specifically bind clinically applicable compounds remains to be explored.

More promising RNA switches were recently proposed based on small‐molecule–mediated alternative splicing to regulate the expression of therapeutic proteins.^[^ ^121^, ^122^ ^]^ Most human genes undergo alternative splicing, a process that yields multiple protein isoforms from the same gene, thus contributing to the diversity of the human proteome. Exerting control over alternative splicing enables the opportunity to engineer novel proteins by either incorporating or skipping protein‐coding exons (Figure 5g). Initial attempts to develop mammalian synthetic RNA devices relying on exon skipping mediated by the tetracycline aptamer showed very limited dynamic ranges.^[^ ^123^, ^124^ ^]^ An alternative splicing‐based RNA switch leveraged a splicing modifier drug (LMI070), currently in clinical trials for the treatment of spinal muscular atrophy, as a trigger for modulating the inclusion or exclusion of a synthetic exon during mRNA splicing.^[^ ^121^ ^]^ By inserting the Kozak sequence and start codon of the target transgene into a splicing site that is responsive to LMI070, protein translation can be controlled. In the default splicing pathway, the start codon is removed and translation is not initiated (Figure 5h). Drug treatment promotes the inclusion of the AUG‐containing pseudoexon in a dose‐dependent manner, which allows translation of the mRNA and production of the target protein. These riboswitches provide greater activation ratios (over 100‐fold) than those based on aptazymes, and despite being slightly larger (over 500 nucleotides for the shorter module), they remain considerably shorter than any transcriptionally regulated switch. Their compatibility with AAV‐based delivery was demonstrated in mouse studies, which showcased their ability to temporally regulate the expression of reporter and therapeutic proteins. Although the switches were optimized to function at low drug concentrations to minimize their impact on other endogenous splicing events, quantifying any residual off‐target effects in the human proteome is yet to be addressed.

Collectively, the majority of the available mammalian riboswitches suffer from limitations that hinder their broad applicability and translation into clinical settings. Perhaps applying a potent ligand‐responsive RNA aptamer and simultaneously control both alternative splicing and ribozyme activity modules within the same transcript might work synergistically to afford broader dynamic ranges.^[^ ^122^ ^]^ As optimization efforts continue and a growing repertoire of aptamers and aptazymes becomes available for cellular applications, it is foreseeable that synthetic riboswitches might come to the forefront as valuable tools in the realm of mammalian synthetic biology.

## 7 Synthetic Devices Relying on Post‐Translational Regulation

When employing chemically or physically induced transcriptional or translational switches, the time it takes for the regulated protein and its corresponding transcript to degrade after inhibition of transcription or translation might not align with certain intended applications. To circumvent slow actuation, various approaches have been developed to directly control proteins at the post‐translational level using small molecules. In this section, we will explore methods that offer broad applicability across a wide range of proteins.

### 7.1 Protein Destabilization Systems

**Figure 6:**
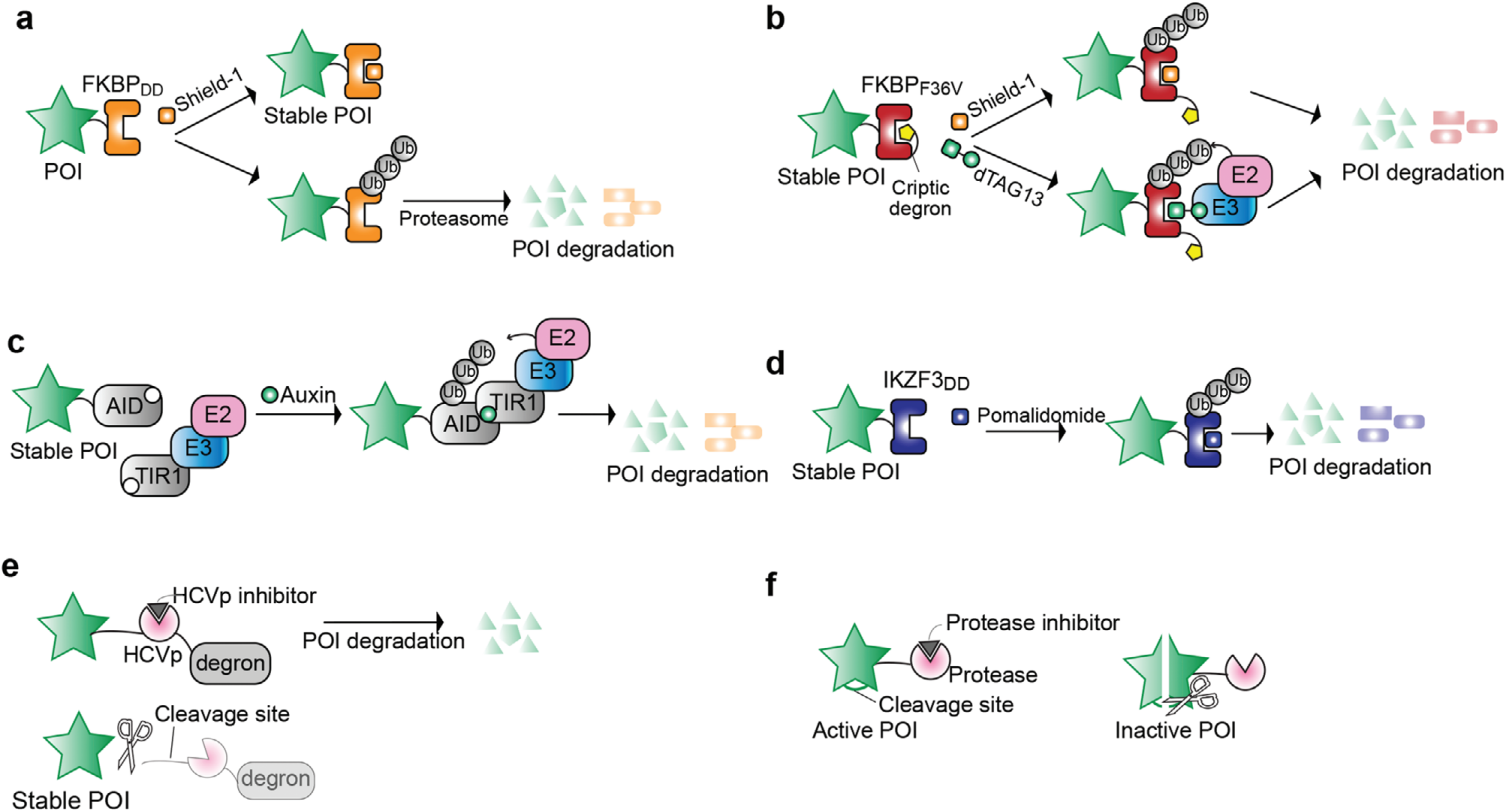
Protein switches for remote control of protein activity. a–d) Small‐molecule–switchable destabilizing domains. a) The small molecule Shield‐1 stabilizes an FKBP‐derived degron fused to a POI, increasing the stability of the fusion protein. b) POIs fused to domains with cryptic degrons, which are unmasked by Shield‐1, or by a heterobifunctional molecule (dTAG13) which localizes the POI with components of the ubiquitin complex, are targeted for degradation in the presence of these molecules. c) The plant‐derived auxin‐inducible degradation (AID) system. POIs fused to an AID degron interact with the auxin receptor transport inhibitor response 1 (TIR1) in the presence of auxin. As TIR1 interacts with the endogenous mammalian ubiquitylation system, the POI is subsequently ubiquitinated (Ub), leading to its rapid degradation by the proteasome. d) POIs fused to a small degron derived from the lymphocyte transcription factor IKZF3 are targeted to degradation in the presence of pomalidomide analogs. e,f) Inhibitable protease‐based switches. e) The SMASh approach. The HCVp and a degron attached to a POI through a HCVp cleavage sequence, are self‐excised by default, rendering the POI functional. In the presence of a protease inhibitor, the degron remains attached, prompting POI degradation. f) POIs fused to an inhibitable protease and harboring a protease cleavage site in an exposed loop can be regulated in response to a protease inhibitor.

Conditional inactivation of proteins can be achieved through the integration of destabilizing domains (DD) (also known as degrons) that can be controlled in response to specific signals.^[^ ^125^, ^126^ ^]^ One of the first general strategies for achieving small‐molecule–mediated control over protein stability capitalizes on destabilizing domains derived from the FKBP protein, which are capable of conferring ligand‐dependent stability when fused to proteins of interest.^[^ ^126^ ^]^ Employing a combination of random mutagenesis and fluorescence‐activated cell sorting (FACS), thousands of variants of the compact FKBP domain (comprising 107 residues) were generated, and a few isolated mutants were capable of significantly diminishing the fluorescence emitted by an attached YFP in the absence of the ligand (rapamycin analogs SLF* or Shield‐1), while significantly augmenting it when the ligand was introduced (**Figure** **6**a). Notably, the FKBP(L106P) variant emerged as the most potent destabilizer, and the stability of the attached protein could be rescued in the presence of the ligand Shield‐1. The efficacy of FKBP‐derived DDs has been demonstrated across a spectrum of proteins localized in the cytoplasm, nucleus, and plasma membrane. While this system operates in an “on‐mode,” with ligand administration stabilizing the protein, the same research group subsequently developed an “off‐mode” system, where the addition of the ligand leads to protein degradation.^[^ ^127^ ^]^ This approach involves the integration of a 19‐amino–acid degron sequence between FKBP and the target protein, which does not affect the stability of the protein when the Shield‐1 ligand is absent. However, when present, Shield‐1 tightly associates with FKBP, displacing the degron and prompting degradation of the fusion protein (Figure 6b). A more efficient off‐mode method couples the mutant FKBP(F36V) attached to the same 19‐aa degron with synthetic heterobifunctional small molecules (called dTAGs) which induce the dimerization of FKBP(F36V) with components of E3 ubiquitin ligase complexes, either CRBN^[^ ^25^ ^]^ or von Hippel–Lindau (VHL^[^ ^26^ ^]^). Through this dTAG system, a diverse array of target proteins could be robustly degraded with the dTAG molecules (formed by covalently linking Shield‐1 to either a CRBN‐binding or a VHL‐binding ligand) functioning effectively in the lower nanomolar range. Most of the target proteins could be substantially degraded within 1 h of treatment. These features underscore the inherent versatility of the platform. In a different application, the abundance of dCas9 fused to a histone deacetylase 4 (HDAC4) could be tuned by a dTAG molecule which dimerizes the attached FKBP(F36V)‐based destabilization domain with CRBN, thereby controlling dCas9‐HDAC4 degradation.^[^ ^128^ ^]^ This approach was effective for controlled, inducible, and reversible gene repression at various targeted loci, in a dTAG‐dependent manner.

Two additional methods for chemically stabilizing proteins capitalize on unstable variants derived from *E. coli* dihydrofolate reductase (ecDHFR)^[^ ^129^ ^]^ or the ligand binding domain of the mammalian estrogen receptor (ER50),^[^ ^130^ ^]^ which were engineered to display drug‐dependent stability. Like the FKBP degron, they were established through error‐prone PCR and FACS, using as stabilizers trimethoprim for ecDHFR (trimethoprim has low affinity for the mammalian DHFR enzyme) and the small molecules CMP8 and 4‐OHT, which have high affinity for the mutant ER (ER50) but not for the wild‐type ER. When POIs are fused to these domains, the entire protein fusions are degraded, unless the respective ligands are present.^[^ ^129^, ^130^ ^]^ These methods have been successfully combined to achieve orthogonal activation of diverse endogenous genes through degron‐regulated dCas9‐based transcription factors.^[^ ^131^ ^]^ Evolved components have been reported over the years. For instance, new ecDHFR variants were established exhibiting significantly enhanced basal proteasomal turnover, while showing increased dynamic range in the presence of the stabilizing small molecule.^[^ ^132^ ^]^

The auxin‐inducible degron (AID) system utilizes plant hormones from the auxin class to regulate the stability of target proteins.^[^ ^125^ ^]^ This system entails tagging target proteins with an AID degron originating from the IAA17 protein, coupled with co‐expression of the auxin receptor transport inhibitor response 1 (TIR1) of the plant *A. thaliana* or *Oryza sativa*. In the presence of the natural auxin indole‐3‐acetic acid (IAA), TIR1 binds to AID‐tagged proteins, facilitating their degradation via the cellular protein degradation machinery (Figure 6c). The AID system operates in an OFF mode, as the addition of the ligand leads to protein degradation. By using CRISPR/Cas9‐based homologous recombination, the AID tag (68 aa residues) has been introduced into endogenous genes, causing the corresponding proteins to experience rapid degradation after the addition of auxin to the culture medium.^[^ ^133^ ^]^ Recent advances have further refined the AID system by incorporating a mutant receptor (TIR1[F74G]) and a modified ligand (5‐phenyl‐IAA). Added benefits include reduced leaky degradation of POIs when the ligand is absent, reduced effective concentrations of the ligand (in the lower micromolar range), and significantly faster degradation rates compared to the original AID system.^[^ ^134^ ^]^ Nevertheless, the need to co‐express the accessory receptor is a drawback in comparison to the other methods outlined in this section.

Another approach to conditionally modulate protein abundance via degradation is based on the immunomodulatory drug pomalidomide (and its analogs), which target the lymphocyte transcription factor IKZF3 for degradation by an E3 ubiquitin ligase complex. A recent study identified a small IKZF3‐derived degron (25 aa residues) that could effectively direct recombinant proteins for degradation upon exposure to pomalidomide, in a dose‐dependent manner (Figure 6d).^[^ ^135^ ^]^

### 7.2 Protease‐Based Systems

Alternative approaches for modulating protein levels by means of exogenous signals involve the utilization of sequence‐specific viral proteases and their inhibitors, functioning as either “on” or “off” protein switches. The genome of many viruses encodes a polyprotein with an embedded protease that cleaves the polyprotein at specific sites to yield mature viral proteins. Given the critical role of these proteases in the viral life cycle, numerous viral protease inhibitors have been developed for therapeutic applications.^[^ ^136^ ^]^ For instance, protease inhibitors have been part of antiviral combination therapies against human immunodeficiency virus (HIV) and hepatitis C virus (HCV). The HCV NS3/4A protease (HCVp) cleaves the viral polyprotein at four sites during virus maturation and the sequence EDVVPC/SM has been identified as its preferred substrate.^[^ ^33^ ^]^ After the TEVp, for which small‐molecule inhibitors are still not available, HCVp has been the most commonly employed protease to create protein switches in mammalian cells.^[^ ^30^, ^31^, ^84^, ^137^ ^]^

The small‐molecule–assisted shutoff (SMASh) approach attaches a degron and HCVp through a protease cleavage sequence to the protein of interest.^[^ ^137^ ^]^ At baseline, the protease and degron are self‐excised, rendering the protein fully functional (Figure 6e). However, upon introduction of the protease inhibitor, the degron remains attached to newly synthesized protein molecules, rapidly prompting their degradation. SMASh was recently compared side‐by‐side with the degron systems based on FKBP‐dTAG, AID, ecDHFR, and ER50, to conditionally control dCas9 abundance.^[^ ^128^ ^]^ For consistency, all degrons were placed at the *N*‐terminus of dCas9. The FKBP and AID systems, which like SMASh induce degradation upon ligand addition, outperformed all the others in terms of minimizing degradation leakiness, maximizing degradation efficiency, and achieving higher fold‐changes between the stabilized and destabilized conditions. Notably, SMASh exhibited slower kinetics, likely because protease inhibitors can only influence newly synthesized proteins, as mature proteins will have already excised the degradation tag.

Various single‐chain protein switches operating in an “on” mode have been developed using either HCVp or the main protease (Mpro) of SARS‐Cov2. Mpro has also been a target for antiviral drug development, with the compound nirmatrelvir (PF‐07321332) exhibiting potential in clinical studies.^[^ ^138^, ^139^ ^]^ Mpro cleaves the viral protein at 11 sites, and a screening process identified TTVRLQSGFRKM as the most efficiently cleaved sequence.^[^ ^8^, ^140^ ^]^ The cleavage sites are strategically placed within the structure of the POI, between two functional domains, or in exposed loops, and the protease is either attached to the *N*‐ or *C*‐terminus, or next to the cleavage site.^[^ ^8^, ^30^, ^31^ ^]^ In contrast to SMASh, these switches inherently maintain the POI in an inactive state due to protease cleavage (Figure 6f). Upon exposure to bioavailable and clinically safe protease inhibitors, the cleavage process is inhibited, thereby preserving the functionality of the POIs. An important characteristic of such one‐component protein switches is their compact design, which results in a small genetic footprint. These switches have demonstrated versatility in controlling the activity of various proteins, including transcription factors based on natural^[^ ^8^ ^]^ and artificial DNA binding domains.^[^ ^84^ ^]^

As previously illustrated in the context of chimeric receptors with orthogonal downstream cascades, the TEV protease has emerged as a commonly employed molecular part of mammalian synthetic gene circuits.^[^ ^109^, ^141^, ^142^, ^143^ ^]^ The activity of TEV has been controlled mainly indirectly through strategies such as inducing dimerization of its split fragments,^[^ ^141^, ^142^ ^]^ or employing cascades with other inhibitable proteases.^[^ ^143^ ^]^ The identification of TEV inhibitors would significantly enhance its applicability and utility for externally controlled protein switches.

## 8 Engineered Cell Therapies

The advance of mammalian synthetic biology has primarily been motivated by the pursuit of improved therapeutic cells. To enhance the efficacy and safety profiles of engineered cell therapies, multiple layers of regulation can be assembled into higher‐order therapeutic programs, triggered by disease biomarkers and/or external stimuli. In this section, we discuss how the molecular components and regulatory systems outlined above have been employed to design next‐generation CAR‐T cells for cancer therapy and how other therapeutic cells are being engineered to tackle diverse diseases.

### 8.1 Regulatable CAR‐T Cells for Cancer Treatment

T cells are engineered with a CAR to sense cancer biomarkers and elicit downstream cytotoxic responses. Multiple generations of CARs have been created to stimulate the optimal combination of intracellular signaling, T cell activation and T cell persistence. There are currently six approved CAR‐T cell products for the treatment of blood malignancies, four of them based on anti‐CD19 CARs and two targeting the B‐cell maturation antigen.^[^ ^144^ ^]^ Although serious side‐effects such as cytokine storms and neurotoxicity can occur,^[^ ^145^ ^]^ many patients have achieved complete remission upon treatment with CAR‐T cells.^[^ ^146^ ^]^ The positive outcomes in B cell malignancies stem from the abundant and consistent expression of lineage antigens primarily found on B cells, and the fact that depletion of normal B cells does not cause significant harm to patients. However, the extension of CAR‐T cells to solid tumors faces constraints due to the limited availability of tumor‐specific surface antigens. While efforts have been made to target antigens overexpressed in tumors, their expression at low abundance in healthy tissues can still result in severe, life‐threatening toxicity.^[^ ^147^, ^148^ ^]^ The identification of additional antigens exhibiting sufficient differential expression levels for safe targeting is crucial for extending the applicability of CAR‐T cells to solid tumors.

**Figure 7:**
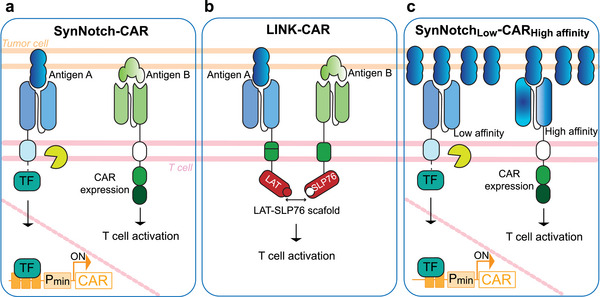
Regulation of CAR‐T cell activation by input signals from the tumor microenvironment. a) SynNotch‐CAR. SynNotch recognizes antigen A and triggers the expression of a conventional CAR against antigen B. b) LINK‐CAR. Split CAR design consisting of two different antigen‐binding scFvs, each fused to one of the signaling proteins LAT and SLP‐76, which form a scaffold upon binding of the two antigens for signal propagation and T cell activation. c) Antigen density recognition circuit. A synNotch receptor detects an antigen with low affinity and induces expression of a high‐affinity CAR for the same antigen.

Extensive research efforts have been directed toward incorporating logic gates into next‐generation receptors to simultaneously detect multiple antigens, with the aim of enhancing the distinction between tumor and normal tissues. Various studies have leveraged synNotch receptors to equip T cells with Boolean logic in order to better tackle a broad range of tumors.^[^ ^4^, ^52^, ^53^, ^54^ ^]^ In these studies, T cells are engineered with a synNotch receptor that recognizes a priming antigen, with its activation subsequently inducing the transcription of a CAR‐targeting a second antigen (**Figure** **7**a). Apart from improving tumor specificity, infused synNotch‐CAR‐T cells exhibit enhanced persistence and potency in vivo compared to T cells constitutively expressing the CAR. Indeed, the dynamic regulation of CARs prevents chronic signaling that would otherwise lead to accelerated T cell exhaustion.^[^ ^53^, ^54^ ^]^ Nonetheless, while the application of AND‐gated synNotch‐CAR‐T cells has shown reduced toxicity when antigen‐expressing healthy cells are physically separated from tumor cells, the presence of cancer cells expressing the priming antigen alongside healthy cells expressing only the second antigen still leads to off‐tumor toxicity.^[^ ^52^ ^]^ This is because the presence of both antigens on the same cell is not a requirement; once the infused T cells are induced to express the CAR, then co‐localized healthy cells expressing only the antigen for the CAR will also be killed.

This issue is absent in the AND‐gated CAR platform known as LINK.^[^ ^149^ ^]^ The two downstream signaling molecules LAT and SLP76, which are both essential for signal propagation and T cell functionality, are used instead of the standard intracellular co‐stimulatory and CD3z signaling domains (Figure 7b). These proteins are fused to different membrane‐bound antigen‐targeting domains. T cell activation is only triggered upon engagement of both antigens and subsequent co‐localization of LAT and SLP76, which form a scaffold for signal propagation. To ensure a robust AND logic gate, a series of targeted mutations were introduced, including the deletion of binding sites for the adaptor protein GADS in the LAT and SLP‐76 proteins, effectively reducing single‐antigen leakiness. In a tumor mouse model, LINK CAR‐T cells successfully eradicated dual‐antigen expressing tumor cells, sparing single‐positive healthy cells, whereas mice treated with synNotch‐CAR‐T cells experienced toxicity and did not achieve the same favorable outcome.

Alternatively, T cells can be engineered in a manner that ensures their activation solely upon encountering a certain antigen density threshold on the surface of target tumor cells^[^ ^150^ ^]^ (Figure 7c). This can be achieved through the implementation of a positive‐feedback circuit involving the utilization of a low‐affinity synNotch receptor for a tumor‐associated antigen (TAA), which controls the expression of a high‐affinity CAR for the same TAA. T cells equipped with this circuit exhibited precise discrimination between target cells with regular HER2 expression and cancer cells with significantly elevated HER2 levels, in both in vitro and in vivo settings.

**Figure 8:**
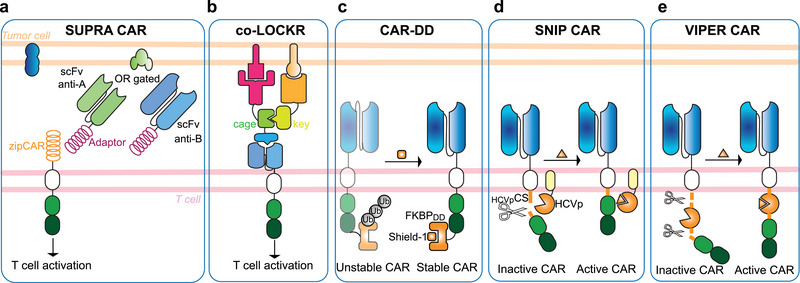
Remote regulation of CARs. a) The split, universal, and programmable CAR (SUPRA‐CAR) has a leucine zipper in the extracellular domain (zipCAR) and requires the administration of adaptor molecules consisting of tumor‐targeting scFvs (against two different antigens for OR logic) fused to a complementary leucine adaptor. b) The Co‐LOCKR system consists of cage and key fusion proteins that bind to two tumor‐associated antigens in target cells. Once the cage and key bind their cognate antigens, a peptide hidden in the cage protein is released, allowing recognition by CAR‐T cells. c) The CAR‐DD has a small‐molecule–switchable destabilizing domain (FKBP_DD_) attached at its *C*‐terminus, which induces rapid degradation in the absence of a stabilizing drug (Shield‐1). d) The signal neutralization by an inhibitable protease (SNIP) CAR approach relies on a standard CAR bearing a HCVp cleavage sequence downstream of the transmembrane (TM) domain, and co‐expression of HCVp fused to a TM domain. e) The VIPER CAR contains HCVp flanked by cleavage sites between the co‐stimulatory domains. In the absence of protease inhibitors (such as grazoprevir), the SNIP and VIPER CARs are inactivated by the protease, while in the presence of grazoprevir, the CARs remain intact and functional.

While the above Boolean logic systems operate based on input signals originating from the tumor environment, making them inherently self‐regulated, the ability to exert external control over implanted cell therapies remains highly attractive in clinical settings. Indeed, CAR‐T cells have also been equipped with logic systems that allow adjustable regulation of T cell activity through exogenous signals.^[^ ^12^, ^13^ ^]^ One such approach is the split, universal and programmable (termed SUPRA) CAR, which operates based on two elements: a universal leucine zipper as the extracellular domain of a CAR, and a soluble tumor‐targeting scFv attached to a leucine adaptor (**Figure** **8**a).^[^ ^13^ ^]^ In the SUPRA CAR design, T cells are activated only when the adaptor fusion protein binds both the tumor antigen and the CAR via the zipper interface. The SUPRA CAR‐T cells can easily be targeted to different antigens without further genetic manipulation, simply by providing a new tumor‐targeting adaptor molecule. Additionally, to counteract antigen escape, OR‐gated tumor killing can be accomplished by introducing two distinct adaptor molecules, each recognizing a different tumor‐specific antigen.^[^ ^13^ ^]^ While SUPRA CAR offers considerable flexibility, it does come with the drawback that activity regulation relies on a protein, which requires delivery by injection, has a relatively short pharmacokinetic half‐life in vivo, and is more limited in terms of achieving effective tumor penetration.

The co‐LOCKR system presents another approach to accomplish combinatorial antigen targeting to improve the specificity of CAR‐T cells, again relying on externally supplied protein components (Figure 8b).^[^ ^12^ ^]^ The underlying logic functions are executed through a set of computationally designed adaptor proteins that interact with one another and modulate binding to the CAR in response to the presence of target antigens. The “cage” and “key” proteins, fused to two different antigen‐targeting DARPins, are designed to interact when co‐localized at the surface of a target cell by the antigen‐binding domains. Within the cage protein there is a hidden peptide capable of binding to and activating the CAR. Upon the binding of key and cage proteins, a conformational change occurs that exposes this peptide, thereby enabling CAR activation. The co‐LOCKR strategy has been successfully integrated into CAR designs to target up to three different antigens expressed on cancer cells. However, the practical application of this approach is constrained by considerations such as the pharmacokinetics and potential immunogenicity associated with the synthetic proteins, as in the case of SUPRA CARs.

Other strategies to improve the therapeutic outcome of engineered T cells rely on post‐translational regulation of the CAR‐through small‐molecule–dependent receptor degradation.^[^ ^151^, ^152^ ^]^ Weber et al. proposed a drug‐regulatable CAR design in which the destabilizing domain (DD) FKBP is attached to the *C*‐terminus of the CAR (Figure 8c).^[^ ^151^ ^]^ This enabled control of CAR protein levels by exposure to the small molecule shield‐1 that binds to the DD, thereby preventing CAR degradation. Induction of “resting periods” achieved by transiently reducing CAR expression prevented therapeutic T cells from undergoing the characteristic phenotypic changes associated with exhaustion. This aligns with the outcomes from the synNotch‐CAR‐T cell studies highlighted earlier.^[^ ^53^, ^54^ ^]^

Two recent studies proposed regulated CARs based on HCVp and its clinically approved inhibitors.^[^ ^84^, ^153^ ^]^ In both systems, the CAR is inactivated at baseline by protease cleavage, and activated upon grazoprevir administration, showing a low level of leaky activity and a wide dynamic range. The SNIP system integrates an HCVp cleavage site between the CAR‐transmembrane and signaling domains and co‐expresses the HCVp in trans attached to the cell membrane (Figure 8d).^[^ ^153^ ^]^ The efficacy and safety of SNIP was extensively demonstrated in several tumor mouse models. In an on‐target off‐tumor ROR1 toxicity model, the SNIP CARs could be finely tuned by adjusting grazoprevir dosing, thereby sparing healthy cells expressing low levels of ROR1, while effectively eliminating ROR1‐expressing tumor cells. Conversely, the VIPER CAR features a single fusion protein that incorporates HCVp positioned between cognate cleavage sites and situated between the intracellular co‐stimulatory domains (Figure 8e).^[^ ^84^ ^]^ Dual‐input–regulated CARs were built by combining protease‐based and DD‐based approaches in a split architecture. This was achieved by fusing the intracellular signaling domain CD3 to one antigen‐targeting domain (anti‐Her2 receptor) and the co‐stimulatory domain to a second antigen‐targeting domain (anti‐Axl receptor), linked respectively to the HCVp or DD regulation system. Separate control over these domains using specific drugs enabled modulation of AND‐gated CAR activity, although there remains room for improvement, as some basal killing was observed in the presence of only one drug.^[^ ^84^ ^]^

Collectively, switches that permit temporal control of CARs activated by multiple TAA show promise for enhancing efficacy, specificity, and diminishing toxicity. They offer superior antitumor efficacy relative to conventional CAR‐T cells owing to delayed exhaustion (enhanced T cell persistence). We expect that these next‐generation CAR‐T cell therapies will be more widely deployed in the near future.

### 8.2 Cell‐Based Therapies for Diverse Chronic Diseases

Genetically engineered human cells have also been explored as therapeutic agents for an array of diseases other than cancer, such as diabetes,^[^ ^6^, ^7^, ^9^, ^14^, ^18^, ^21^, ^36^, ^50^, ^154^ ^]^ gouty arthritis,^[^ ^155^ ^]^ methicillin‐resistant *Staphylococcus aureus* infection,^[^ ^156^ ^]^ and psoriasis,^[^ ^157^ ^]^ among others.^[^ ^2^ ^]^ While these studies have demonstrated promise in preclinical settings, the cells have not yet been developed for clinical use. Many of these initiatives have relied either on implantation of engineered HEK293 cells encapsulated in immune‐protective micro‐ or macro‐encapsulation devices, or on the delivery of the genetic circuit components to host cells by naked DNA transfection or viral vector transduction.^[^ ^6^, ^14^, ^18^, ^154^ ^]^ An important decision when considering ex vivo‐modified therapeutic cells for implantation is selection of the appropriate cell type. While immortalized cell lines offer advantages for initial proof‐of‐concept studies, transfer of the optimized synthetic gene circuits to medically relevant cell sources is imperative for translational purposes.

Mesenchymal stem cells (MSCs) have emerged as a promising candidate for cell‐based therapies, given their remarkable immunomodulatory attributes and tissue‐regenerative potential. Extensive clinical exploration in more than a thousand trials registered in the ClinicalTrials.gov database, have established a robust safety profile, leading to regulatory approval of over 20 MSC‐based therapies worldwide.^[^ ^158^ ^]^ MSCs are easily isolated from several tissues, including bone marrow, adipose tissue, or umbilical cord blood. The capacity to be expanded in vitro and differentiated into several cell types further underscores their attractiveness. Notably, MSCs are immune‐privileged, as confirmed in clinical trials involving allogeneic MSCs that demonstrated excellent tolerance (e.g., ref. [159]). Their regenerative features and functionalities can be further augmented through the integration of the synthetic biology tools outlined above to finely tune their behavior.^[^ ^160^ ^]^ This will accelerate the clinical translation of designer MSCs.

Alternatively, various immune cell types other than T cells exhibit favorable attributes for engineered cell therapies. These include natural killer (NK) cells, non‐canonical T cells such as γδ T cells, B lymphocytes, and others. B cell engineering, in particular, offers unique opportunities for developing new therapeutic approaches for various diseases,^[^ ^161^ ^]^ expanding the scope of T cell‐based therapies. Within the adaptive immune system, B cells are defined by the expression of a membrane‐tethered antibody, known as the B cell receptor (BCR). Upon BCR binding to a matching antigen, coupled with co‐stimulatory cues, B cells undergo proliferation and differentiate into memory B cells and plasma cells. Their ability to stably produce high concentrations of antibodies, combined with their ease of collection from peripheral blood, and suitability for reinfusion into patients, places B cells as an attractive chassis for engineered cell therapies. They have already been used for expression of recombinant antibodies^[^ ^162^ ^]^ and other therapeutic proteins.^[^ ^163^, ^164^ ^]^ Recently, Cheng et al. showed that ex vivo‐engineered and differentiated human plasma cells have the potential for long‐lived in vivo protein secretion.^[^ ^164^ ^]^

Many of the desirable features of engineered B cells require that the cells maintain responsiveness to antigen, which can be achieved if the engineered antibodies maintain a BCR format. Genome editing allows reprograming of BCRs through site‐specific insertion of custom antibody components at appropriate sites within the Ig locus. Several groups have now demonstrated site‐specific CRISPR/Cas9‐mediated integration of recombinant genes to replace the endogenous BCR.^[^ ^165^ ^]^ These studies have largely leveraged ex vivo techniques,^[^ ^165^, ^166^ ^]^ but direct in vivo cell modification with AAVs^[^ ^167^ ^]^ or adenoviral vectors (AV)^[^ ^168^ ^]^ has also been achieved.

The potential of engineered B cells has been extensively explored to fight HIV‐1 infection. Prior studies have demonstrated the ex vivo integration of broadly neutralizing antibodies (bnAbs) genes into the Ig locus of B cells using CRISPR‐Cas9.^[^ ^162^, ^169^ ^]^ Upon transfer to mice, the modified B cells effectively participated in humoral immune responses, secreting the corresponding bnAbs.^[^ ^162^, ^169^ ^]^ More recently, Nahmad et al. used two AAV6 vectors to pack the CRISPR/Cas9 components targeting the Ig heavy chain locus and the anti‐HIV‐1 bnAb 3BNC117, and delivered them intravenously for in vivo B cell engineering in mice.^[^ ^167^ ^]^ B cells were effectively modified in vivo and differentiated into 3BNC117‐expressing cells that exhibited memory responses.

The feasibility of random integration of a synthetic gene circuit into B cells through lentivirus transduction was recently assessed.^[^ ^170^ ^]^ The circuit consisted of constitutive expression of antigen‐targeting BCR and a reporter protein controlled by a region of the promoter of the nuclear receptor NR4A1, which is known to be activated upon BCR stimulation.^[^ ^171^ ^]^ Although increased NR4A1‐driven reporter protein expression was observed in cells treated with antigen‐coated beads, the fold‐activations in relation to treatment with uncoated beads were very low. Improved promoter designs might provide enhanced performance.

In contrast to T cells, B cells do not possess inherent cytotoxic capabilities, making them more suitable candidates for cellular therapies targeting metabolic conditions characterized by elevated levels of particular biomarkers. B cells could be genetically engineered with biomarker sensing abilities, enabling them to secrete therapeutic proteins that counteract elevated biomarker levels, thereby establishing a closed‐loop approach to managing disease states. Encouraging results from a pioneering clinical trial assessing the safety and tolerability of adoptively transferred donor B cells in patients over a 4 months follow‐up period, in which no adverse responses were observed,^[^ ^172^ ^]^ represent a step forward in this direction.

## 9 Conclusions and Perspectives

While engineered cell‐based therapies offer transformative potential to restore cellular functions in patients with incurable diseases, many remaining challenges must be addressed to ensure widespread clinical success across a range of different diseases. One significant hurdle is the manufacturing challenges and high production costs associated with current CAR‐T cell therapies, largely arising from the necessity to custom‐engineer each patient's own cells ex vivo post‐collection of peripheral blood mononuclear cells. The first approved CAR‐T therapy Kymriah costs nearly half a million USD per patient^[^ ^173^ ^]^ and the timeline from collection to re‐infusion extended over 3 to 4 weeks. A promising path currently under clinical exploration involves allogeneic T cells from healthy donors, known as “off‐the‐shelf” therapies, which would not require custom engineering and would speed up the manufacturing process, thus having the potential to expedite treatment for severely ill patients. To decrease the risk of graft‐versus‐host disease reactions, allogeneic T cells have been gene‐edited in single^[^ ^174^, ^175^ ^]^ or multiple loci.^[^ ^176^ ^]^ While the disruption of the endogenous TCR α chain (TRAC locus) prevents donor CAR‐T cells from recognizing patient alloantigens, components of the major histocompatibility complex class I can also be disrupted to avoid recognition of the donor CAR‐T cells by T cells of the patient, thereby enhancing immune compatibility. The advent and evolution of CRISPR editing platforms have enabled the establishment of multi‐gene–edited therapeutic cells for “off‐the‐shelf” use.^[^ ^176^ ^]^ Advances in prime editing technology will allow the development of safer therapeutic cells, particularly by avoiding double‐strand breaks, thereby minimizing the consequences of unwanted indels associated with standard CRISPR/Cas9.^[^ ^177^, ^178^ ^]^ The first clinical trials using gene‐edited allogeneic CAR‐T cells have been initiated.^[^ ^179^ ^]^ Next‐generation allogeneic cell sources will be derived from gene‐edited immunocompatible pluripotent stem cells, which have unlimited expansion potential and can be differentiated in vitro into all cell types.^[^ ^180^, ^181^ ^]^

Alternative paths to overcome the cost of therapy and the manufacturing difficulties could involve directly administering genetic circuits into patients, by‐passing the isolation and/or (re‐)infusion of cells.^[^ ^182^, ^183^ ^]^ Progress in virus capsid design and screening for targeting viral vectors to specific tissues and cell types holds the potential to enhance the precision of gene delivery.^[^ ^184^ ^]^

Although numerous manufacturing and regulatory hurdles will need to be addressed, exciting approaches are currently under development to increase the efficacy and scope of engineered cell‐based therapies while improving their safety. We believe this will encourage the adoption of this technology for multiple applications, opening up new therapeutic frontiers.

## Conflict of Interest

The authors declare no conflict of interest.
